# Association of area‐level mortgage denial and guideline‐concordant non‐small‐cell lung cancer care and outcomes in the United States

**DOI:** 10.1002/cam4.6921

**Published:** 2024-01-11

**Authors:** Qinjin Fan, S. M. Qasim Hussaini, Lauren C. J. Barrow, Josephine L. Feliciano, Craig E. Pollack, K. Robin Yabroff, Leticia Nogueira

**Affiliations:** ^1^ Department of Surveillance and Health Equity Science American Cancer Society Atlanta Georgia USA; ^2^ Sidney Kimmel Comprehensive Cancer Center Johns Hopkins Hospital Baltimore Maryland USA; ^3^ Department of Health Policy and Management Johns Hopkins Bloomberg School of Public Health Baltimore Maryland USA; ^4^ Johns Hopkins School of Nursing Baltimore Maryland USA

**Keywords:** guideline‐concordant care, mortgage denial, non‐small‐cell lung cancer, social determinants of health

## Abstract

**Background:**

Racial and socioeconomic disparities in receipt of care for non‐small‐cell lung cancer (NSCLC) are well described. However, no previous studies have evaluated the association between mortgage denial rates and receipt of timely and guideline‐concordant care for NSCLC and patient outcomes.

**Methods:**

We identified individuals ≥18 years diagnosed with NSCLC between 2014 and 2019 from the National Cancer Database. Using the Home Mortgage Disclosure Act database, we calculated the proportion of denied home loans to total loans at the zip‐code level and categorized them into quintiles. Our outcomes included receipt of guideline‐concordant care based on clinical and pathologic stage at diagnosis and the National Comprehensive Cancer Network guidelines, time from surgery to chemotherapy initiation, and overall survival.

**Results:**

Of the 629,288 individuals diagnosed with NSCLC (median age 69; IQR 61–76 years, 49.1% female), 47.8% did not receive guideline‐concordant care. Residing in areas with higher mortgage denial rates and lower income was associated with worse guideline‐concordant care overall (aRR = 1.28; 95% CI = 1.25–1.32) and for each cancer treatment modality, worse receipt of timely chemotherapy (aHR = 1.14; 95% CI = 1.11–1.17) and worse overall survival (aHR = 1.21; 95% CI = 1.19–1.22), compared with residing in areas with the lowest mortgage denial rate and highest income.

**Conclusions:**

Area‐level mortgage denial rate was associated with worse receipt of timely and guideline‐concordant NSCLC care and survival. This highlights the critical need to understand and address systemic practices, such as mortgage denial, that limit access to resources and are associated with worse access to quality cancer care and outcomes.

## 1 INTRODUCTION

Lung cancer is the second most common cancer diagnosed and the leading cause of cancer‐related deaths in the United States (US), with an estimated 238,340 new cases and 127,070 deaths in 2023.^1^ Understanding modifiable factors associated with access to quality lung cancer care can inform efforts to improve patient outcomes.

Clinical practice guidelines compile available evidence and expert consensus in treatment recommendations that improve survival and quality of life. However, not all individuals diagnosed with cancer in the US receive guideline‐concordant cancer care.^2^ Among the multiple factors that influence receipt of quality cancer care and outcomes, social determinants reflect interconnected social structures shaped by policies and practices that determine the distribution of resources.^3^ Historical and current mortgage lending practices shape resource distribution between communities. For example, mortgage lending bias is strongly related to disparities in housing access as well as other neighborhood health determinants including access to resources, socioeconomic status, and built environment features.^4^ Therefore, it constitutes an important modifiable social determinants of health^5^ and critical upstream structural determinants of health which is essential to support comprehensive patient care and address disparities in numerous health outcomes including cancer.^6^, ^7^, ^8^

Studies have documented persisting racial and ethnic discrimination in mortgage lending, limiting the housing options available to individuals from communities targeted for marginalization, a factor that has been associated with disparities in cancer outcomes due to reduced access to quality food, transportation, health care, and preventive services, educational and employment opportunities, recreation, and disproportionate exposure to environmental hazards.^9^, ^10^, ^11^, ^12^ In an effort to identify and address discriminatory policies and practices in mortgage lending, the Home Mortgage Disclosure Act (HMDA) was passed in 1975 requiring the public reporting of lending practices. Previous studies have used HMDA data to identify wide variation across neighborhoods in their rates of mortgage denials.^13^, ^14^ However, these studies evaluating the association between mortgage denial and cancer mortality were limited to two racialized groups (Black and White), individuals 65 years or older at the time of diagnosis, individuals residing in metropolitan areas or in a single state.^11^, ^12^ In contrast, in this study, we investigated the association between area‐level mortgage denial rates, a modifiable social determinant of health, and access to quality cancer care and outcomes among individuals of all ages and racialized groups who were newly diagnosed with NSCLC and resided in metropolitan, suburban, or rural areas in all states.

## 2 METHODS

### 2.1 Study population

Individuals newly diagnosed with NSCLC between January 1, 2014 and December 31, 2019 were selected from the National Cancer Database (NCDB), a nationwide hospital‐based cancer registry jointly sponsored by the American College of Surgeons and the American Cancer Society. The NCDB includes more than 1500 Commission on Cancer accredited facilities and more than 70% of individuals newly diagnosed with cancer in the US.^15^

Individuals were excluded if they were <18 years of age, had a concomitant diagnosis or history of other malignant tumors, or were diagnosed with noninvasive adenocarcinoma. Individuals diagnosed at death or with missing information on residential zip code at diagnosis, area‐level median household income, date of diagnosis, or date of last contact were also excluded. Cohort selection is shown in Figure S1. The present study was deemed exempt from review by the Institutional Review Board.

### 2.2 Outcome measures

Eligibility and receipt of guideline‐concordant care was defined according to National Comprehensive Cancer Network (NCCN) guidelines version 3.2022—March 16, 2022, using the 7th and 8th edition of the American Joint Commission on Cancer (AJCC) clinical and pathological stage at diagnosis. Guideline‐concordant care treatment modalities included surgery or radiation, lymph node evaluation, neoadjuvant chemoradiation, and any chemotherapy or immunotherapy (Table S1). The single palliative care measure in NCDB identifies any care provided in an effort to palliate or alleviate symptoms and is a summary across modalities (e.g., radiation therapy, systemic therapy) and is not directly comparable with other treatment data. Therefore, receipt of palliative care was not included in the analysis. The NCCN guidelines for NSCLC cancer,^16^ did not change for the examined treatment modalities during the study period. Individuals who received all treatments for which they were eligible were considered to have received guideline‐concordant care. Other outcomes were time to adjuvant chemotherapy initiation (number of days between surgery date and chemotherapy initiation date, date of last contact or death, or study end, whichever came first) and overall survival (interval between age at diagnosis and age at death, last contact, or study end, whichever came first) through December 31, 2019.

### 2.3 Mortgage denial rates

We obtained mortgage denial data from the HMDA database (2014–2019),^11^ which is publicly available and collects mandatorily reported information on mortgage lending practices for primary residences, including the location for which a mortgage was requested (census tract) and loan status (approval/denial). Because refinancing is an important resource for people experiencing financial hardship, data for both purchase and refinance home loans, and all types of homes (on‐site built or manufactured) were included. The mortgage denial rates were estimated as the proportion of denied home loans to the total of completed home loans and were categorized into quintiles, where level 1 (≤7.9) is the lowest denial rate, and level 5 (>15.1) is the highest denial rate. In order to combine the census tract‐level mortgage denial rates with the zip code‐level NCDB data, we redistributed the census tract‐level HMDA data to zip code level using the Department of Housing and Urban Development (HUD)'s Office of Policy Development and Research zip code to census tract crosswalk file.^17^, ^18^ Denial rates were not calculated where the count of loan events during the 6‐year study period was less than 10 to ensure stable estimates, and individuals residing in areas where number of loans was less than 10 were excluded.^19^

### 2.4 Study population characteristics

Individual‐level demographic measures included: age at diagnosis, sex, and race and ethnicity. Race and ethnicity were ascertained from patients' medical records. Although cancer registries use standardized data items and codes for assigning race and ethnicity from medical records, the initial collection of this information by healthcare facilities and providers and the methods used to codify this information in medical records are not standardized. Patient comorbidities were measured with the modified Charlson and Deyo comorbidity index (0, 1, or ≥2).^20^ Individual‐level socioeconomic measures were health insurance coverage type, categorized into uninsured, private insurance, Medicare Public, Medicare Private, and Other Public (Medicaid, Medicare with Medicaid eligibility, Veterans Affairs, and Indian/Public Health Service). Zip code‐level median household income and educational attainment reflect area‐level socioeconomic conditions.

### 2.5 Statistical analyses

Descriptive statistics and comparisons by area‐level mortgage denial rate categories were conducted using the Pearson Chi‐square tests. The association of area‐level mortgage denial rates and nonreceipt of guideline‐concordant care overall and by each treatment modality (i.e., not receiving surgery/radiation, ≥10 lymph nodes evaluation, neoadjuvant chemoradiation, any chemotherapy or immunotherapy) among eligible patients based on the pathological and clinical stage at diagnosis was estimated with generalized estimating equations. Risk ratios and predicted probabilities (adjusting for age and sex) were estimated. The association between mortgage denial rate and time from surgery to adjuvant chemotherapy initiation was examined with a multivariable Cox proportional hazard model. The association of mortgage denial rate and overall survival following NSCLC diagnosis was evaluated with the multivariable Cox proportional hazard models using age as the time scale. The proportional hazards assumption was tested by examining Schoenfeld residuals and we did not observe any violations of the assumption. All models were adjusted for individual demographic characteristics (age at diagnosis and/or sex) and accounted for clustering within zip codes. For each outcome measure, we evaluated its association with area‐level mortgage denial rate by area‐level income (2012 zip‐code level median household income). We presented the results using the most privileged group (individuals residing in the lowest mortgage denial rate and highest income areas) as the reference group and reported the type III chi‐square test for multiplicative interactions between area‐level income and mortgage denial levels.

In sensitivity analysis, mortgage denial rates were categorized into quartiles. We chose not to include race/ethnicity in the adjusted models to avoid incorrectly assigning race (a social construct that should only be used as a proxy for exposure to racism) as a risk factor.^21^ Instead, we evaluated the interaction between individual racialized groups and area‐level mortgage denial rate using the most privileged group (individuals racialized as White residing in low mortgage denial rate areas) as the reference group and reported the type III chi‐square test for multiplicative interactions between racialized groups and mortgage denial rate in the Supplemental Tables. All statistical analyses were performed with SAS9.4 software (SAS Institute, Cary, NC), and all tests were two‐sided at a significance level of 0.05.

## 3 RESULTS

A total of 629,288 individuals newly diagnosed with NSCLC, who resided in 39,211 different zip code areas were included in the study. Median age at diagnosis was 69 years (IQR 61–76 years); 308,635 (49.1%) were women and 320,653 (50.9%) were men; 81.1% were non‐Hispanic White, 11.6% were non‐Hispanic Black. Individuals residing in high mortgage denial rate areas were more likely to be male, aged 45–64 years, lack health insurance coverage, be diagnosed with stage IV disease, and have more comorbid conditions (Table 1). Non‐Hispanic White individuals were more likely to reside in areas with low mortgage denial rates than non‐Hispanic Black and Hispanic individuals. Area‐level educational attainment and median income were highly correlated with mortgage denial rates. For example, within the lowest median income category, 64.1% and 3.5% of individuals resided in areas with the highest and lowest mortgage denial rates, respectively. Within the highest income category, 4.9% and 57.1% of individuals resided in areas with the highest and lowest mortgage denial rates, respectively. Although correlated, there was substantial variation of mortgage denial rates within each income category, and the ranges overlap across income categories. For instance, the interquartile range of mortgage denial rate is 8.2–12.1 for areas with incomes between $50 and $60 k, 7.0–10.6 for areas with incomes between $70 and $80 k, and 6.9–10.2 for areas with incomes exceeding $80 k (Figure S2).

**TABLE 1:** Characteristics of individuals newly diagnosed with non‐small‐cell lung cancer in the years 2014–2019 by mortgage denial level^a^.

	Area‐level mortgage denial rate category												
	Overall	% Calcs	1‐Low	% Calcs	2	% Calcs	3	% Calcs	4	% Calcs	5‐High	% Calcs	*p*‐value^e^
Number of patients	629,288	100.00	125,853	20.00	125,838	20.00	125,883	20.00	125,866	20.00	125,848	20.00	
Median age (IQR)	69 (61–76)		70 (62–77)		69 (62–77)		69 (61–76)		69 (61–76)		68 (60–75)		
Age													
18–44	9877	1.57	2187	1.74	1859	1.48	1906	1.51	1861	1.48	2064	1.64	<0.0001
45–64	209,183	33.24	37,652	29.92	40,212	31.96	41,673	33.10	42,788	33.99	46,858	37.23	
65–75	220,761	35.08	43,688	34.71	44,112	35.05	44,533	35.38	44,529	35.38	43,899	34.88	
≥75	189,467	30.11	42,326	33.63	39,655	31.51	37,771	30.01	36,688	29.15	33,027	26.24	
Sex													
Male	320,653	50.95	61,442	48.82	62,603	49.75	64,623	51.33	65,056	51.68	66,929	53.18	<0.0001
Female	308,635	49.05	64,411	51.18	63,235	50.25	61,260	48.67	60,810	48.32	58,919	46.82	
Race/ethnicity													
Hispanic	22,011	3.50	2434	1.93	2786	2.21	3579	2.84	4343	3.45	8869	7.05	<0.0001
Non‐Hispanic Asian and Pacific Islander	19,472	3.09	4397	3.49	3959	3.15	4339	3.45	3533	2.81	3244	2.58	
Non‐Hispanic Black	72,700	11.55	5086	4.04	7136	5.67	8988	7.14	14,124	11.22	37,366	29.69	
Non‐Hispanic Other^b^	5024	0.80	992	0.79	896	0.71	863	0.69	911	0.72	1362	1.08	
Non‐Hispanic White	510,081	81.06	112,944	89.74	111,061	88.26	108,114	85.89	102,955	81.80	75,007	59.60	
Comorbidity^c^													
0	357,389	56.79	72,920	56.66	71,298	56.43	71,033	56.11	70,629	56.82	71,509	56.80	<0.0001
1	156,154	24.81	30,581	24.81	31,222	25.01	31,489	25.14	31,637	24.81	31,225	24.85	
≥2	115,745	18.39	22,352	18.53	23,318	18.56	23,361	18.75	23,600	18.37	23,114	18.35	
Insurance coverage type													
Uninsured	4652	2.49	1980	1.57	2564	2.04	3025	2.40	3478	2.76	4652	3.70	<0.0001
Private	27,356	25.19	35,099	27.89	33,722	26.80	31,993	25.41	30,346	24.11	27,356	21.74	
Medicare Public	33,819	27.63	35,044	27.85	34,769	27.63	34,833	27.67	35,409	28.13	33,819	26.88	
Medicare Private	29,842	28.09	39,154	31.11	37,497	29.80	35,925	28.54	34,345	27.29	29,842	23.71	
Other Public^d^	28,198	15.22	13,011	10.34	15,611	12.41	18,296	14.53	20,635	16.39	28,198	22.41	
Missing	1981	1.38	1565	1.24	1675	1.33	1811	1.44	1653	1.31	1981	1.57	
Area‐level median household income													
<40 k	152,726	24.27	4448	3.53	9800	7.79	18,256	14.50	39,516	31.40	80,706	64.13	<0.0001
40–50 k	157,902	25.09	17,136	13.62	30,235	24.03	41,623	33.06	42,301	33.61	26,607	21.14	
50–63 k	142,711	22.68	32,436	25.77	37,338	29.67	35,439	28.15	25,135	19.97	12,363	9.82	
≥63 k	175,949	27.96	71,833	57.08	48,465	38.51	30,565	24.28	18,914	15.03	6172	4.90	
Area‐level educational attainment (% < high school graduates)													
≥21%	116,501	18.51	1407	1.12	5488	4.04	16,990	13.50	29,397	23.36	63,219	50.23	<0.0001
13%–20.9%	180,421	28.67	9719	7.72	31,154	22.93	44,107	35.04	50,028	39.75	45,413	36.09	
7%–12.9%	206,705	32.85	47,164	37.48	59,465	43.78	48,009	38.14	37,800	30.03	14,267	11.34	
<7%	125,661	19.97	67,563	53.68	29,731	21.89	16,777	13.33	8641	6.87	2949	2.34	
Stage at diagnosis													
Stage I	158,359	25.17	33,876	26.92	32,527	25.85	31,774	25.24	31,193	24.78	28,989	23.04	<0.0001
Stage II	56,951	9.05	11,426	9.08	11,494	9.13	11,523	9.15	11,451	9.10	11,057	8.79	
Stage III	123,778	19.67	23,130	18.38	24,595	19.54	24,666	19.60	25,409	20.19	25,978	20.64	
Stage IV	266,728	42.38	52,951	42.07	52,688	41.87	53,176	42.24	53,088	42.18	54,825	43.56	
Incomplete stage	23,472	3.73	4470	3.55	4534	3.60	4744	3.77	4725	3.75	4999	3.97	

^a^ Data source: National Cancer Database.

^b^ Non‐Hispanic Other includes non‐Hispanic American Indian, Aleutian, or Eskimo (includes all indigenous populations of the Western hemisphere) and Other (for multiracial).

^c^ Other Public insurance includes Medicaid, Medicare with Medicaid eligibility, Veterans Affairs, and Indian/Public Health Service.

^d^ Comorbidity is categorized into three levels (0, 1, or ≥2).

^e^ *p*‐values are for Chi‐square tests.

### 3.1 Nonreceipt of guideline‐concordant treatment

**FIGURE 1:**
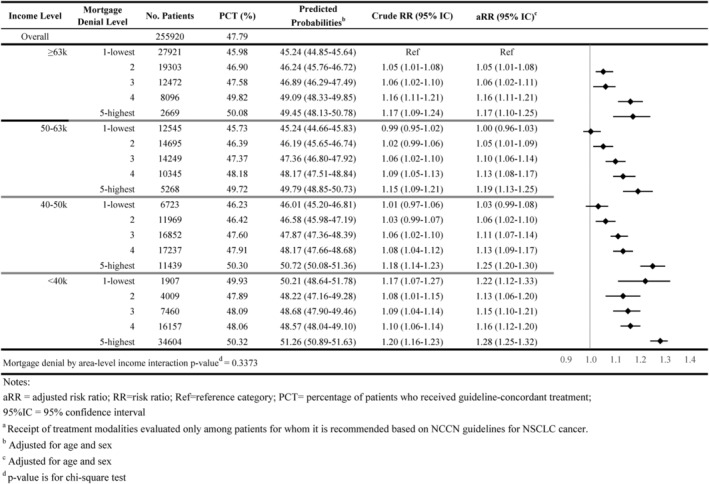
Association of area‐level mortgage denial rates and non‐guideline‐concordant NSCLC care by area‐level income: overall guideline‐concordant care^a^.

**FIGURE 2:**
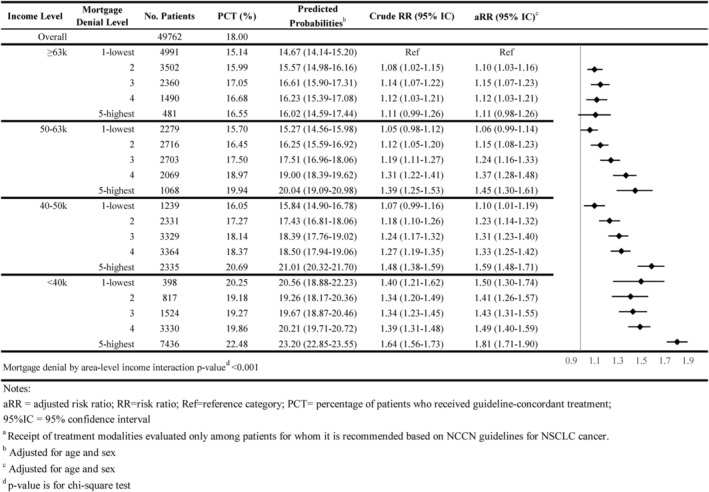
Association of area‐level mortgage denial rates and non‐guideline‐concordant NSCLC care by area‐level income: surgery^a^.

**FIGURE 3:**
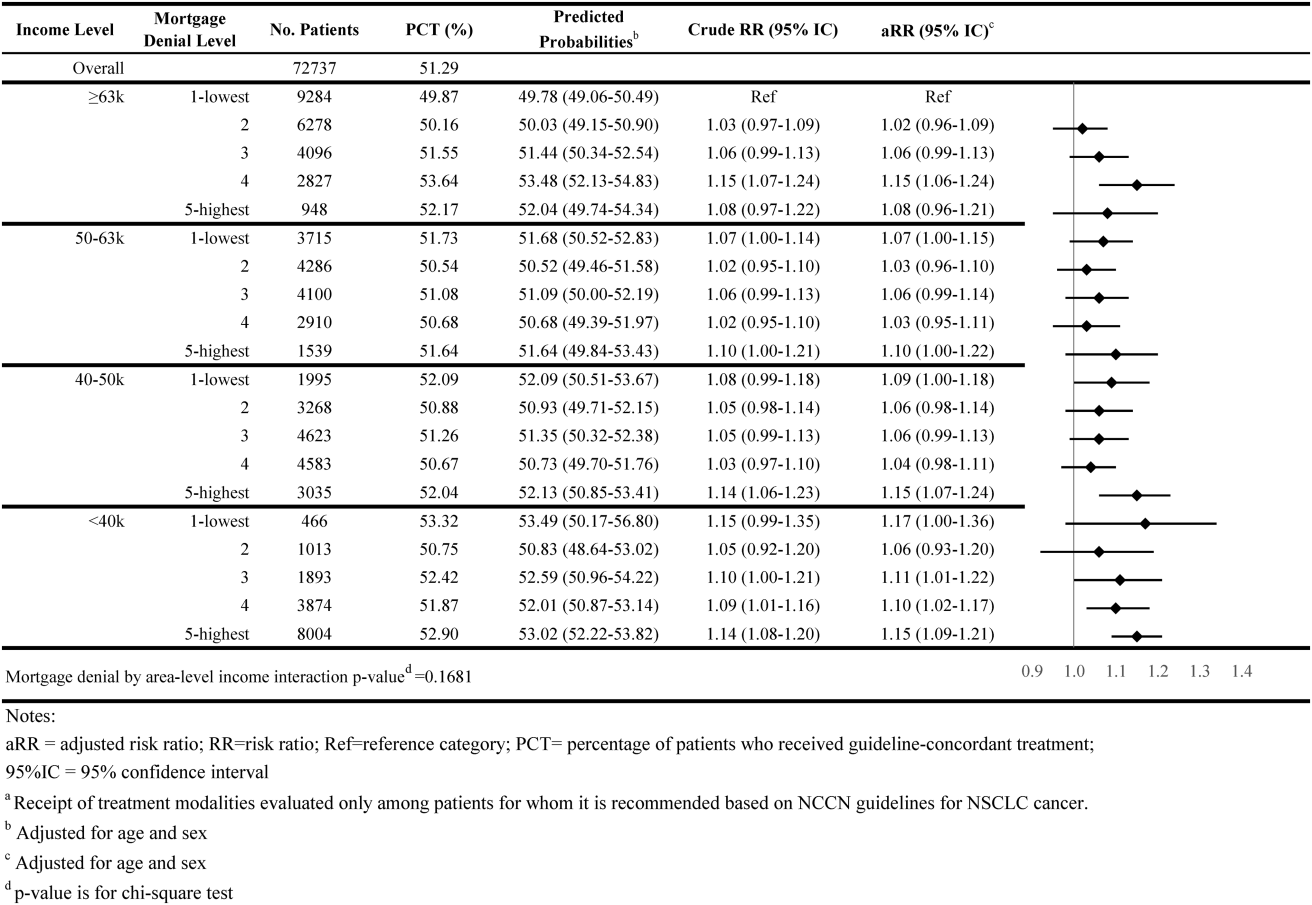
Association of area‐level mortgage denial rates and non‐guideline‐concordant NSCLC care by area‐level income: ≥10 lymph nodes evaluated^a^.

**FIGURE 4:**
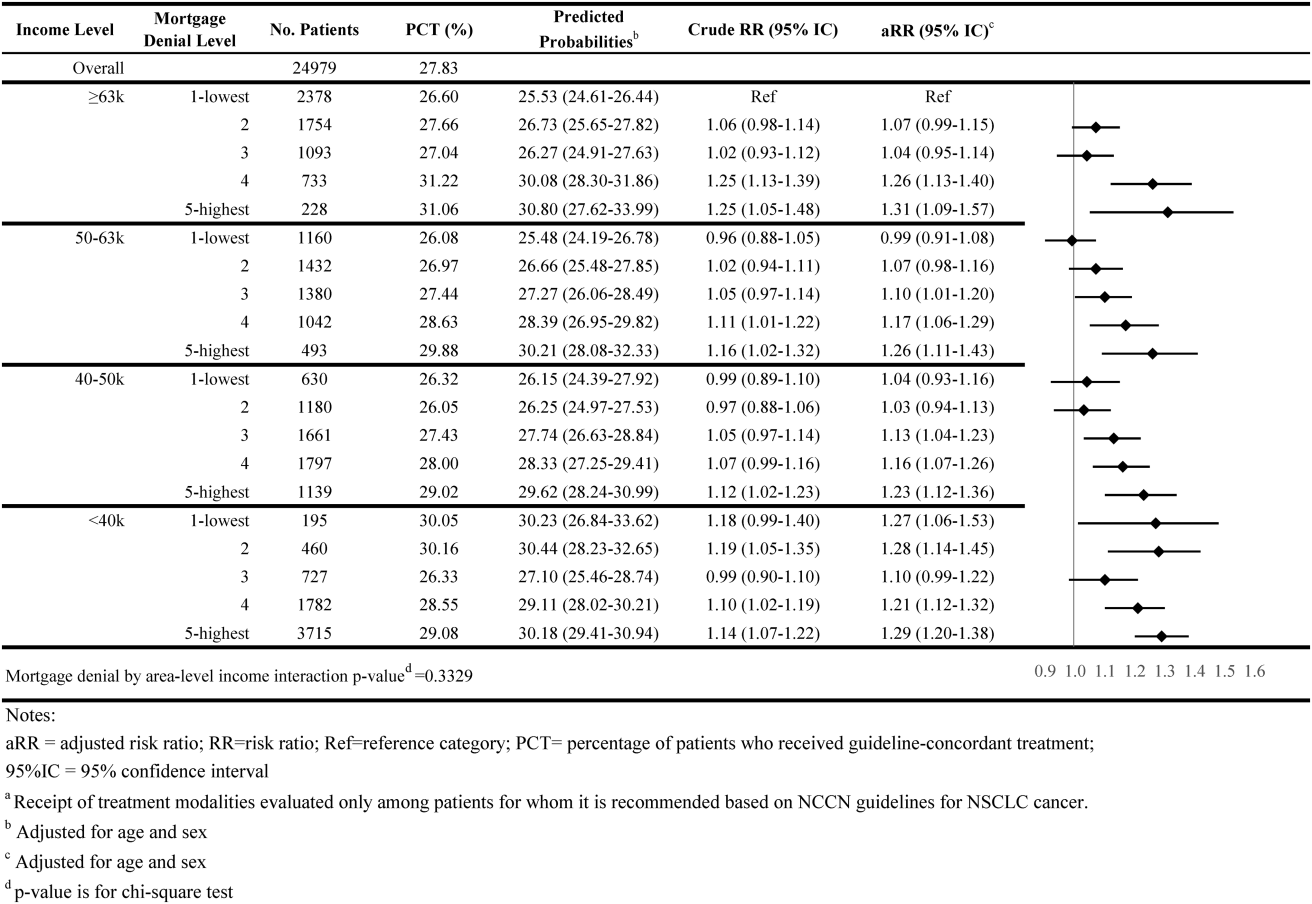
Association of area‐level mortgage denial rates and non‐guideline‐concordant nsclc care by area‐level income: neoadjuvant chemoradiation^a^.

**FIGURE 5:**
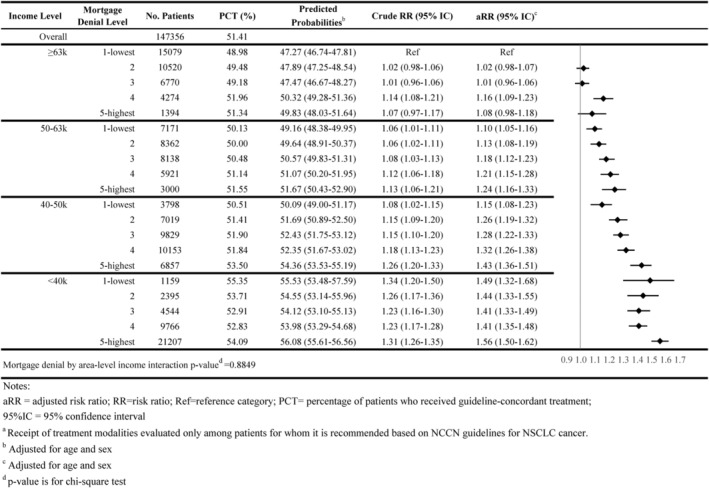
Association of area‐level mortgage denial rates and non‐guideline‐concordant NSCLC care by area‐level income: chemotherapy or immunotherapy^a^.

As shown in Figures 1, 2, 3, 4, 5, 47.8% of all individuals diagnosed with NSCLC between 2014 and 2019 did not receive guideline‐concordant care. Among eligible individuals, 18.0% did not receive surgery, 51.3% did not have ≥10 lymph nodes evaluated, 27.8% did not receive neoadjuvant chemoradiation, and 51.4% did not receive chemotherapy or immunotherapy.

Individuals residing in high mortgage denial rate areas were more likely to not receive guideline‐concordant care compared with individuals residing in low denial rate areas for all income levels. For example, 51.3% of individuals residing in the highest mortgage denial rate and lowest income areas did not receive guideline‐concordant care (adjusted Risk Ratio [aRR] = 1.28; 95% CI = 1.25–1.32) compared with 45.2% of individuals who resided in lowest denial rate and highest income areas, after adjusting for age at diagnosis and sex (Figure 1). The association between mortgage denial rate and nonreceipt of guideline‐concordant treatment was consistent across all treatment modalities, also following a dose–response relationship. Individuals residing in the highest mortgage denial rate and lowest income areas had 1.81 times the risk of not receiving surgery or radiation therapy (aRR = 1.81; 95% CI = 1.71–1.90), 1.15 times the risk of not having at least 10 lymph nodes evaluated (aRR = 1.15 95% CI = 1.09–1.21), 1.29 times the risk of not receiving neoadjuvant chemoradiation (aRR = 1.29; 95% CI = 1.20–1.38), and 1.56 times the risk of not receiving receive chemotherapy or immunotherapy (aRR = 1.56; 95% CI = 1.50–1.62) than individuals residing in the lowest mortgage denial rate and highest income areas (Figures 2, 3, 4, 5).

**FIGURE 6:**
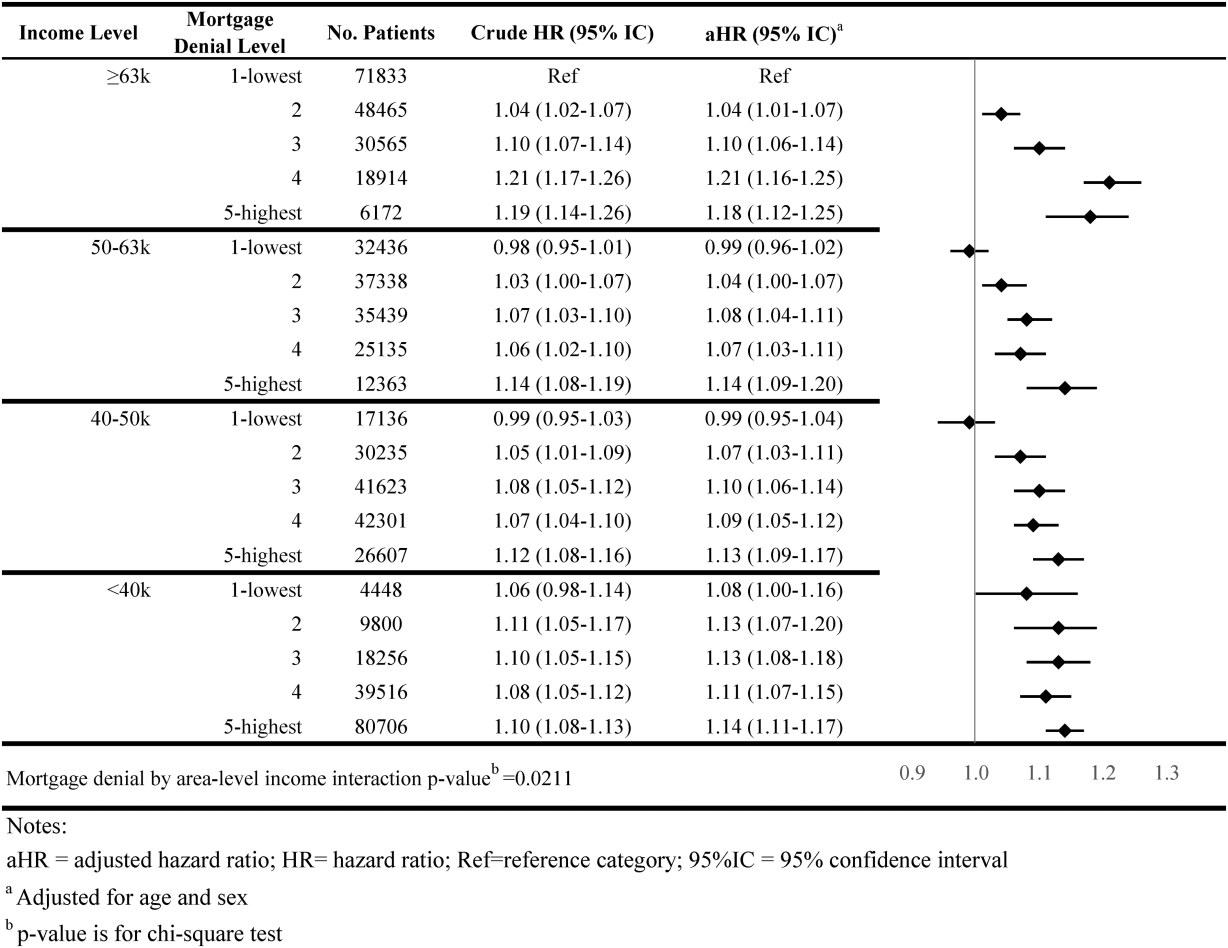
Association of area‐level mortgage denial rates and time from surgery to chemotherapy initiation by area‐level income.

**FIGURE 7:**
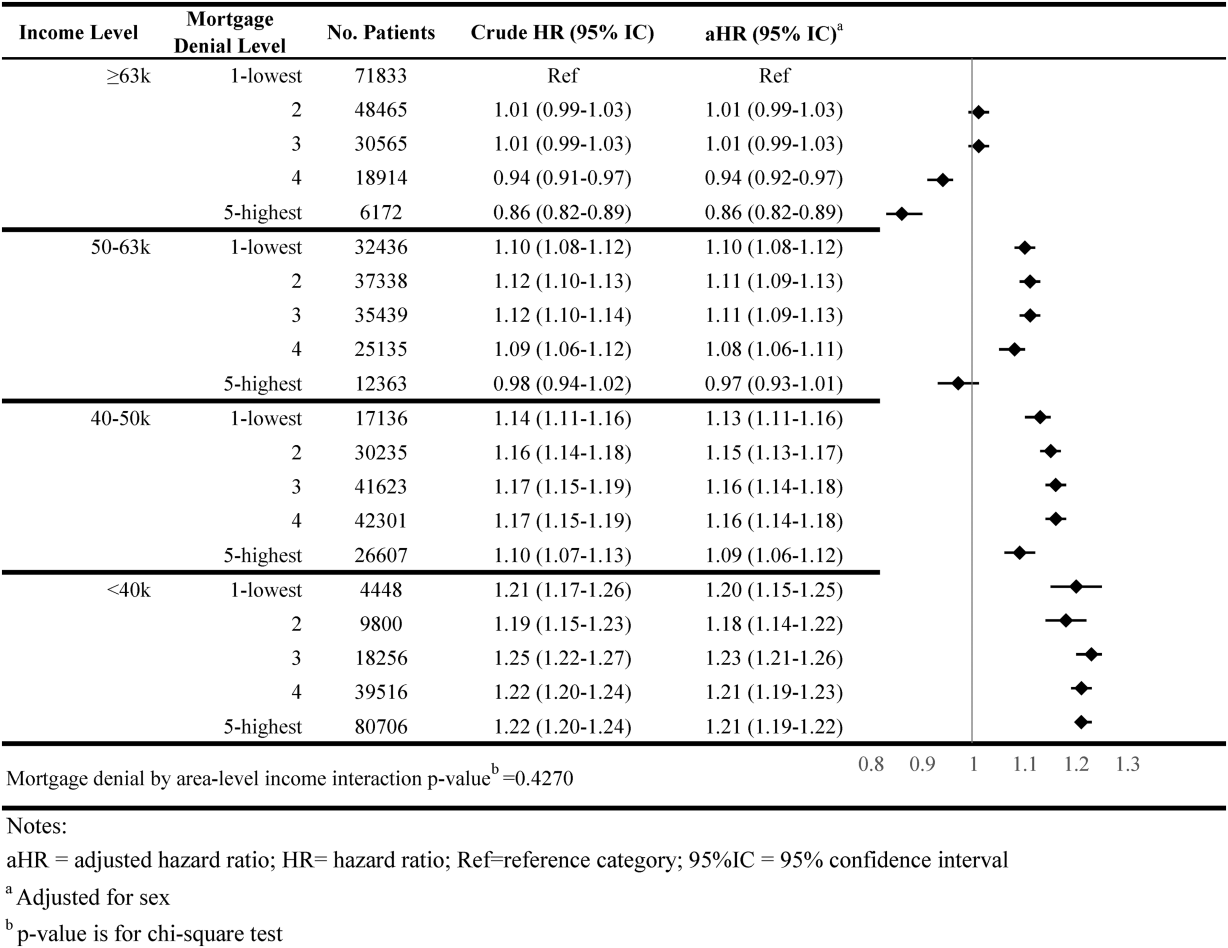
Association of area‐level mortgage denial rates and overall survival (age as time scale) by area‐level income.

### 3.2 Time‐to‐treatment initiation

Individuals residing in the highest mortgage denial rates and the lowest income areas had a longer time to chemotherapy initiation compared with those residing in low denial rates and high‐income areas (adjusted Hazard Ratio [aHR] = 1.14; 95% CI = 1.11–1.17), associations followed a dose–response relationship (Figure 6).

### 3.3 Overall survival

Residing in areas with higher mortgage denial rates and lower income was associated with worse survival after adjusting for sex (aHR = 1.21; 95% CI = 1.19–1.22) compared to areas with lower mortgage denial rates and higher income (Figure 7).

The interaction between area‐level income and area‐level mortgage denial rate only showed significance for receipt of surgery and timeliness of adjuvant chemotherapy (*p*‐value < 0.05; Figures 1, 2, 3, 4, 5, 6, 7).

### 3.4 Sensitivity analyses

In sensitivity analysis, using quartiles of mortgage denial rates as the exposure (rather than quintiles of denial rates) did not significantly alter associations with nonreceipt of guideline‐concordant care (Tables S2–S6), timeliness of adjuvant chemotherapy (Table S7), or survival (Table S8).

Further, residing in areas with higher mortgage denial rates was significantly associated with nonreceipt of guideline‐concordant care, worse time to treatment initiation, and worse overall survival, compared to residing in areas with higher mortgage denial rates for all racialized groups. The interaction between the racialized group and area‐level mortgage denial rate was only significant for receipt of neoadjuvant chemoradiation (*p*‐value < 0.05; Tables S9–S15).

## 4 DISCUSSION

In this large, national study we found that residing in areas with high mortgage denial rates was significantly associated with worse receipt of guideline‐concordant care, worse timeliness of adjuvant chemotherapy initiation, and worse overall survival among individuals newly diagnosed with NSCLC in the US. Further, the association between area‐level mortgage denial rate and receipt of recommended treatments followed a dose–response relationship, with an increasing likelihood of not receiving guideline‐concordant care with increasing rates of mortgage denial.

Residing in higher mortgage denial rate areas was associated with worse receipt of guideline‐concordant care overall and for each treatment modality evaluated, including surgery or radiation therapy for individuals diagnosed with early stage disease, evaluation of ≥10 lymph nodes among individuals who received surgery, receipt of neoadjuvant chemoradiation among individuals with clinical evidence of lymph node involvement (cN2 and cN3), and receipt of chemotherapy or immunotherapy among eligible individuals.

Moreover, residing in areas with higher mortgage denial was associated with a longer time to adjuvant chemotherapy initiation compared with residences in low mortgage denial rate areas, across different income levels. Finally, residing in areas with higher mortgage denial rates was associated with poorer survival following NSCLC diagnosis. This finding aligns with one prior study that has reported an association between denial rates and poor breast cancer survival among older women in the US.^13^

Higher mortgage denial rates may reflect systematic disinvestment in ways that reduce access to care for individuals diagnosed with NSCLC. For example, higher mortgage denial rates may lower home values, reduce resources available to fund education, invest in infrastructure and amenities, such as transportation, decrease community wealth, and/or access to resources during times of hardship (in the case of cash‐out refinances). This contributes to worse area‐level social determinants of health, such as limited development of healthcare facilities and transportation in the area, creating barriers to access to quality cancer care.^22^ If fewer providers practice in the area, eligible individuals will then have fewer opportunities to receive recommendations for and referrals to lung cancer screening. Lower rates of lung cancer screening will reduce early detection of NSCLC. Further, systematic disinvestment in specific neighborhoods may limit access to health‐promoting goods and services and increase exposure to factors that harm health, including a higher concentration of alcohol and tobacco outlets and higher levels of stress.

Mortgage denial measures provide additional information about neighborhood structure, opportunities, and development when taken into consideration with other neighborhood measures, such as area‐level income. For example, the structure of the mortgage process provides little incentive for financial institutions to lend to communities with lower incomes and smaller houses. Nonetheless, the present study found that higher mortgage denial rates were associated with worse access to quality care at all income levels.

Previous studies have evaluated different mortgage denial measures and reported that racial bias in mortgage lending practices and structural inequity were associated with disparities in cancer mortality in the US metropolitan areas.^9^, ^10^, ^14^, ^19^ The HMDA was created to address high mortgage denial rates for certain racialized groups, which can impact housing availability and serve as a marker for other forms of structural racism, such as inequitable access to education, employment, and insurance coverage. Consequently, high mortgage denials may have a more detrimental effect on quality cancer care for racialized groups targeted for marginalization than for those from privileged racialized groups.

Finally, though we mapped the exposure (mortgage denial rates) to the zip code, it is possible that some individuals may have directly experienced mortgage denials and other forms of systematic racism resulting in reduced ability to purchase or refinance homes or receiving less favorable rates in ways that impact homeownership and wealth generation. These may limit resources available to offset the financial burden of cancer and further contribute to housing instability in ways that have been associated with discontinuity and gaps in cancer‐related medical care.^6^ Policies aimed at improving access to mortgage lending need to prioritize equitable distribution of resources (to avoid historical discriminatory practices/redlining‐era exacerbation of disparities) and be coupled with consumer protections (to avoid mortgage‐crisis‐era predatory lending and investment schemes), which have detrimental consequences for the entire population.

A major strength of this study is the large number of individuals of all ages and racialized groups residing in metropolitan, suburban, or rural areas in all states, combined with the extensive demographic and clinical characteristics as well as detailed treatment information (including treatment dates) available in the NCDB. Although the Surveillance, Epidemiology and End Results (SEER)‐Medicare database can be used to evaluate receipt of cancer treatment, this database includes only Medicare beneficiaries (the vast majority of whom are ≥65 years of age). Therefore, SEER‐Medicare cannot be used to evaluate associations between mortgage denial rates and quality cancer care and outcomes in younger populations. Finally, results were consistent when using different methods for classifying area‐level mortgage denial rates.

This study has limitations. First, NCDB is a hospital‐based (instead of population‐based) cancer registry, only capturing cases diagnosed or treated in Commission on Cancer–affiliated hospitals. Nonetheless, NCDB captures over 70% of incident cancer cases in the US and facilities collect treatment information even when it is provided outside of the reporting facility, strengthening the generalizability of our findings. Moreover, the demographic and clinical characteristics of cancer patients in the NCDB are comparable to those from population‐based cancer registries.^15^ Second, NCDB does not collect specific chemotherapy agents; therefore, we considered receipt of any chemotherapy as guideline‐concordant. Other limitations of our study include the unavailability of residential history in NCDB, hindering our ability to evaluate the length of exposure and residential mobility. The NCDB only provides zip code‐level information for patients' residential locations, which constitutes the finest geographic granularity available in the data set. Additionally, it is important to note that our study focuses on area‐level social determinant of health, and as such, we did not incorporate individual‐level socioeconomic factors.

## 5 CONCLUSIONS

Area‐level mortgage denial was adversely associated with receipt of timely and guideline‐concordant NSCLC care and survival. Our findings highlight the critical need to understand and address pathways through which modifiable institutional practices, such as area‐level mortgage denial rates, may contribute to household‐level and neighborhoods' resource availability that can adversely affect access to quality cancer care and outcomes.

## AUTHOR CONTRIBUTIONS

**Qinjin Fan:** Conceptualization (lead); formal analysis (lead); investigation (lead); methodology (lead); project administration (equal); validation (equal); visualization (lead); writing – original draft (lead); writing – review and editing (equal). **S. M. Qasim Hussaini:** Validation (equal); writing – review and editing (equal). **Lauren C. J. Barrow:** Validation (equal); writing – review and editing (equal). **Josephine L. Feliciano:** Validation (equal); writing – review and editing (equal). **Craig E. Pollack:** Conceptualization (supporting); validation (equal); writing – review and editing (equal). **K. Robin Yabroff:** Conceptualization (supporting); supervision (equal); validation (equal); writing – review and editing (equal). **Leticia Nogueira:** Conceptualization (supporting); investigation (supporting); supervision (lead); validation (equal); writing – review and editing (equal).

## FUNDING INFORMATION

Housing assistance, Outcomes, Medicare, and SEER (HOMES) (Grant number: R01CA269488).

## Supporting information


Data S1.

## Data Availability

The data used in this study were provided by the American College of Surgeons to researchers at the American Cancer Society under the Limited Use Dataset agreement. Researchers associated with the Commission on Cancer accredited cancer programs can request access to the data through the Participant User Files (https://www.facs.org/quality‐programs/cancer/ncdb/puf) or through Limited Use Dataset agreements.

## References

[1] American Cancer Society . Facts & Figures 2022. 2022. https://www.cancer.org/content/dam/cancer‐org/research/cancer‐facts‐and‐statistics/annual‐cancer‐facts‐and‐figures/2022/2022‐cancer‐facts‐and‐figures.pdf 10.6004/jadpro.2020.11.2.1 PMC7848816 33532112

[2] Clair K , Chang J , Ziogas A , et al. Disparities by race, socioeconomic status, and insurance type in the receipt of NCCN guideline concordant care for select cancer types in California. Journal of Clinical Oncology. 2020;38:7031.

[3] Alcaraz KI , Wiedt TL , Daniels EC , Yabroff KR , Guerra CE , Wender RC . Understanding and addressing social determinants to advance cancer health equity in the United States: a blueprint for practice, research, and policy. CA Cancer J Clin. 2020;70(1):31‐46. doi:10.3322/caac.21586 31661164

[4] Namin S , Xu W , Zhou Y , Beyer K . The legacy of the home Owners' loan corporation and the political ecology of urban trees and air pollution in the United States. Soc Sci Med. 2020;246:112758. 31884239 10.1016/j.socscimed.2019.112758

[5] Bailey ZD , Krieger N , Agénor M , Graves J , Linos N , Bassett MT . Structural racism and health inequities in the USA: evidence and interventions. Lancet. 2017;389(10077):1453‐1463. 28402827 10.1016/S0140-6736(17)30569-X

[6] Fan Q , Keene DE , Banegas MP , et al. Housing insecurity among patients with cancer. J Natl Cancer Inst. 2022;114:1584‐1592. 36130291 10.1093/jnci/djac136 PMC9949594

[7] Fan Q , Nogueira L , Yabroff KR , Hussaini SMQ , Pollack CE . Housing and cancer care and outcomes: a systematic review. J Natl Cancer Inst. 2022;114(12):1601‐1618. 36073953 10.1093/jnci/djac173 PMC9745435

[8] Naik Y , Baker P , Ismail SA , et al. Going upstream—an umbrella review of the macroeconomic determinants of health and health inequalities. BMC Public Health. 2019;19:1‐19. 31842835 10.1186/s12889-019-7895-6 PMC6915896

[9] Beyer KMM , Laud PW , Zhou Y , Nattinger AB . Housing discrimination and racial cancer disparities among the 100 largest US metropolitan areas. Cancer. 2019;125(21):3818‐3827. doi:10.1002/cncr.32358 31287559 PMC6788939

[10] Zhou Y , Bemanian A , Beyer KMMM . Housing discrimination, residential racial segregation, and colorectal cancer survival in southeastern Wisconsin. Cancer Epidemiol Biomarkers Prev. 2017;26(4):561‐568. doi:10.1158/1055-9965.EPI-16-0929 28196847

[11] Bureau CFP . Home mortgage disclosure act data. 2021.

[12] Lynch EE , Malcoe LH , Laurent SE , Richardson J , Mitchell BC , Meier HCS . The legacy of structural racism: associations between historic redlining, current mortgage lending, and health. SSM‐Popul Heal. 2021;14:100793. doi:10.1016/j.ssmph.2021.100793 PMC8099638 33997243

[13] Beyer KMM , Zhou Y , Laud PW , et al. Mortgage lending bias and breast cancer survival among older women in the United States. J Clin Oncol. 2021;39(25), 2749. doi:10.1200/jco.21.00112 34129388 PMC8407650

[14] Collin LJ , Gaglioti AH , Beyer KM , et al. Neighborhood‐level redlining and lending bias are associated with breast cancer mortality in a large and diverse metropolitan area. Cancer Epidemiol Biomarkers Prev. 2021;30(1):53‐60. doi:10.1158/1055-9965.EPI-20-1038 33008873 PMC7855192

[15] Mallin K , Browner A , Palis B , et al. Incident cases captured in the national cancer database compared with those in U.S. population based central cancer registries in 2012–2014. Ann Surg Oncol. 2019;26(6):1604‐1612. doi:10.1245/s10434-019-07213-1 30737668

[16] National T, Cancer C . Non‐small cell lung. *Cancer* 2011.

[17] HUD's Office of Policy Development and Research (PD&R) . HUD USPS zip code crosswalk files. Accessed July 18, 2022. https://www.huduser.gov/portal/datasets/usps_crosswalk.html

[18] Din A , Wilson R . Crosswalking ZIP codes to census geographies: geoprocessing the US Department of Housing & Urban Development's ZIP code crosswalk files. UMBC Department of Geography & Environmental Systems; 2020.

[19] Beyer KMM , Zhou Y , Matthews K , Bemanian A , Laud PW , Nattinger AB . New spatially continuous indices of redlining and racial bias in mortgage lending: links to survival after breast cancer diagnosis and implications for health disparities research. Heal Place. 2016;40:34‐43. doi:10.1016/j.healthplace.2016.04.014 27173381

[20] Klabunde CN , Potosky AL , Legler JM , Warren JL . Development of a comorbidity index using physician claims data. J Clin Epidemiol. 2000;53(12):1258‐1267. 11146273 10.1016/s0895-4356(00)00256-0

[21] Boyd RW , Lindo EG , Weeks LD , McLemore MR . On racism: a new standard for publishing on racial health inequities. Heal Aff Blog. 2020;10:1.

[22] U.S. Department of Health & Human Services . Community health and economic prosperity: engaging businesses as stewards and stakeholders—a report of the surgeon general. U.S. Department of Health and Human Services, Public Health Service, Centers for Disease Control and Prevention Office of the Associate Director for Policy and StrategyAtlanta, GA 2021.

