# Supplementary material for: Association of area‐level mortgage denial and guideline‐concordant non‐small‐cell lung cancer care and outcomes in the United States
Source: Cancer Med. 2024 Jan 11;13(3):e6921. doi: 10.1002/cam4.6921 (PMC10911071; doi:10.1002/cam4.6921)
Supplement: Supplementary file 1 — Data S1. [file CAM4-13-e6921-s001.docx]

## Supplemental Figure and Tables

**Supplementary Figure 1.** Consort diagram for cohort selection


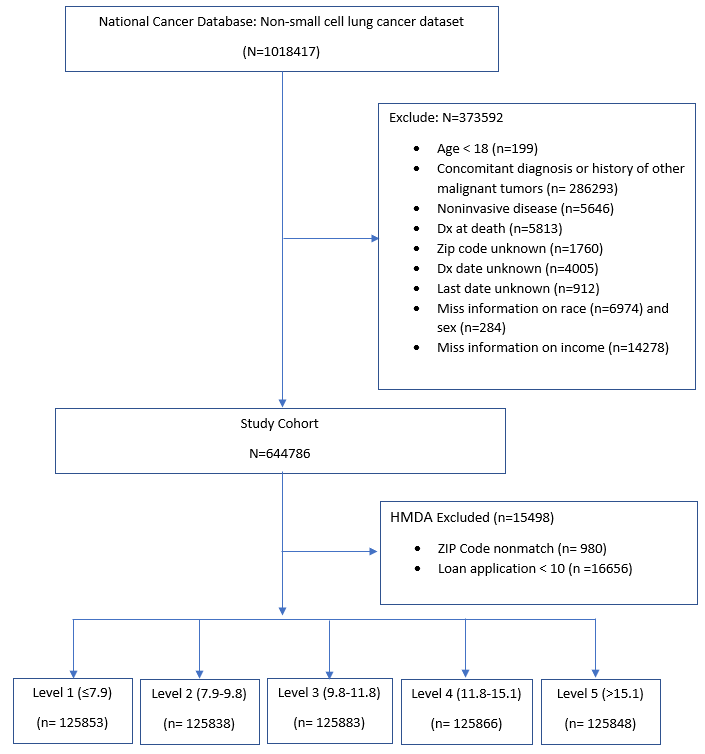


**Supplementary Figure 2.** Box Plot of Area-Level Median Household Income and Mortgage Denial Rate

**
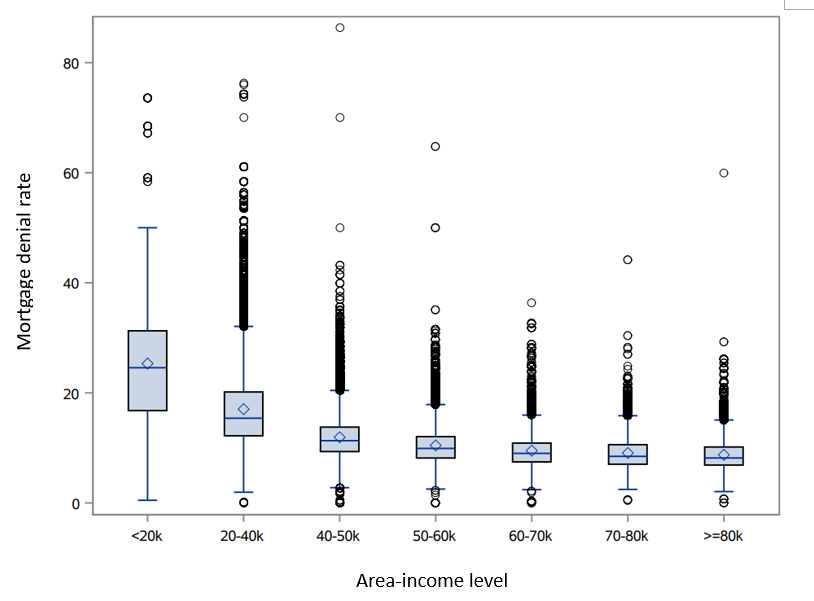
**

|  |
| --- |

**Supplementary Table 1.** Definition of eligibility and receipt of guideline-concordant care for patients diagnosed with Non-Small Cell Lung Cancer, by stage^a^

| **Guideline-concordant** | **Stage** | | | | |
| --- | --- | --- | --- | --- | --- |
|  | **Stage Unknown** | **Stage I** | **Stage II** | **Stage III** | **Stage IV** |
| **Surgery:**  Surgery or radiation therapy initated^b^ | Include*:* All | Include*:* All | Include*:* All | Include:  Patients with any T3-T4, M0 tumors  Exclude:  Patients for whom surgery should not be the first treatment (diagnosed with cN2 and cN3 tumors) | Exclude:  Patients with clinical evidence of metastatic disease (cM1) |
| **10 Lymph Nodes:**  ≥10 regional lymph nodes examined | Exclude:  Patients who did not receive surgery | Exclude:  Patients who did not receive surgery | Exclude:  Patients who did not receive surgery | Exclude:  Patients who did not receive surgery | Exclude:  Clinical or pathologic evidence of metastatic disease |
| **Neoadjuvant Chemoradiation:**  Chemotherapy or radiation therapy initiated before surgery |  |  |  | Include*:* Patients diagnosed with cN2, cN3 tumors | Include*:* Patients diagnosed with cN2, cN3 tumors  Exclude:  Patients with clinical evidence of metastatic disease (cM1) |
| **Any Chemotherapy:**  Chemotherapy or immunotherapy initiated | Include*:* All | Exclude*:* Patients diagnosed with clinical or pathologic T1 or T2 tumors | Exclude*:* Patients diagnosed with clinical or pathologic T1 or T2 tumors | Include*:* All | Include*:* All |

**Notes:**

^a^ NCCN Clinical Practice Guidelines in Oncology (NCCN Guidelines). Non-Small Cell Lung. Cancer. Published online 20112023.avalibale at: https://www.nccn.org/professionals/physician_gls/pdf/nscl.pdf.

**^b^** Radiation therapy is the recommended treatment modality for patients with inoperable tumors. Because NCDB does not collect information on whether the tumor was operable, receipt of either surgery or radiation therapy was considered guideline-concordant.

**Supplementary Table 2.** Sensitivity Analysis Examining Quartiles of Mortgage Denial Rates: Association of Area-Level Mortgage Denial Rate and Non-Guideline-Concordant NSCLC Care by Area-Level Income: Overall Guideline-Concordant Care^a^

| Income level | Mortgage Denial rate | No. Patients | PCT (%) | Predicted Probabilities  (Adjusted for age and sex) | Crude RR (95%IC) | aRR (95%IC)  Adjusted for age and sex | P-value^b^ |
| --- | --- | --- | --- | --- | --- | --- | --- |
| ≥63k | 1-lowest | 76291 | 46.22 | 45.48 (45.13-45.83) | Ref | Ref |  |
|  | 2 | 45850 | 47.10 | 46.45 (45.99-46.90) | 1.04 (1.01-1.07) | 1.04 (1.01-1.08) |  |
|  | 3 | 22078 | 49.26 | 48.57 (47.92-49.22) | 1.13 (1.09-1.17) | 1.13 (1.09-1.18) |  |
|  | 4-highest | 5456 | 50.00 | 49.38 (48.06-50.69) | 1.15 (1.08-1.22) | 1.16 (1.08-1.23) |  |
| 50-63k | 1-lowest | 41362 | 45.91 | 45.51 (45.03-45.99) | 0.99 (0.96-1.02) | 1.00 (0.97-1.03) |  |
|  | 2 | 52668 | 46.60 | 46.59 (46.17-47.01) | 1.02 (0.99-1.05) | 1.05 (1.02-1.08) |  |
|  | 3 | 36201 | 47.90 | 47.92 (47.41-48.43) | 1.07 (1.03-1.10) | 1.10 (1.07-1.14) |  |
|  | 4-highest | 13587 | 49.86 | 49.94 (49.11-50.77) | 1.14 (1.09-1.20) | 1.19 (1.13-1.24) |  |
| 40-50k | 1-lowest | 19162 | 46.80 | 46.71 (46.01-47.41) | 1.03 (0.99-1.07) | 1.05 (1.01-1.10) |  |
|  | 2 | 40554 | 47.11 | 47.35 (46.87-47.84) | 1.04 (1.01-1.07) | 1.08 (1.05-1.12) |  |
|  | 3 | 48195 | 47.90 | 48.20 (47.75-48.64) | 1.07 (1.04-1.10) | 1.12 (1.09-1.15) |  |
|  | 4-highest | 27225 | 49.92 | 50.37 (49.78-50.95) | 1.15 (1.11-1.20) | 1.21 (1.17-1.26) |  |
| <40k | 1-lowest | 4198 | 48.62 | 49.12 (47.62-50.62) | 1.11 (1.02-1.20) | 1.17 (1.07-1.27) |  |
|  | 2 | 11764 | 48.87 | 49.32 (48.43-50.22) | 1.12 (1.06-1.18) | 1.17 (1.11-1.24) |  |
|  | 3 | 28780 | 48.37 | 48.94 (48.36-49.51) | 1.09 (1.05-1.13) | 1.15 (1.11-1.20) |  |
|  | 4-highest | 62098 | 50.48 | 51.49 (51.10-51.88) | 1.20 (1.16-1.23) | 1.29 (1.25-1.33) |  |
| Area-level income level*Denial rate | |  |  |  |  |  | 0.2129 |

Notes:

aRR = adjusted risk ratio; RR=risk ratio; Ref=reference category; PCT= percentage of patients who received guideline-concordant treatment; 95%IC = 95% confidence interval

^a^ Receipt of treatment modalities evaluated only among patients for whom it is recommended based on NCCN guidelines for NSCLC cancer.

^b^ p-value is for chi-square test

**Supplementary Table 3.** Sensitivity Analysis Examining Quartiles of Mortgage Denial Rates: Association of Area-Level Mortgage Denial Rate and Non-Guideline-Concordant NSCLC Care by Area-Level Income: Surgery^a^

| Income level | Mortgage Denial rate | No. Patients | PCT (%) | Predicted Probabilities  (Adjusted for age and sex) | Crude RR (95%IC) | aRR (95%IC)  Adjusted for age and sex | P-value^b^ |
| --- | --- | --- | --- | --- | --- | --- | --- |
| ≥63k | 1-lowest | 41321 | 15.25 | 14.79 (14.43-15.16) | Ref | Ref |  |
|  | 2 | 24202 | 16.50 | 16.07 (15.59-16.54) | 1.10 (1.04-1.16) | 1.11 (1.05-1.17) |  |
|  | 3 | 12064 | 16.90 | 16.47 (15.80-17.15) | 1.13 (1.06-1.22) | 1.13 (1.06-1.21) |  |
|  | 4-highest | 2973 | 16.45 | 15.93 (14.57-17.29) | 1.09 (0.97-1.23) | 1.09 (0.96-1.23) |  |
| 50-63k | 1-lowest | 21861 | 15.84 | 15.49 (14.99-16.00) | 1.05 (0.99-1.11) | 1.06 (1.01-1.13) |  |
|  | 2 | 27098 | 16.90 | 16.89 (16.44-17.34) | 1.14 (1.08-1.20) | 1.18 (1.11-1.24) |  |
|  | 3 | 18564 | 18.46 | 18.48 (17.94-19.02) | 1.26 (1.19-1.34) | 1.31 (1.24-1.39) |  |
|  | 4-highest | 6839 | 19.87 | 19.93 (19.04-20.83) | 1.38 (1.26-1.51) | 1.44 (1.31-1.57) |  |
| 40-50k | 1-lowest | 10135 | 16.84 | 16.72 (15.99-17.46) | 1.13 (1.05-1.21) | 1.16 (1.08-1.24) |  |
|  | 2 | 21072 | 18.00 | 18.16 (17.65-18.67) | 1.22 (1.16-1.30) | 1.28 (1.20-1.35) |  |
|  | 3 | 24591 | 18.54 | 18.76 (18.29-19.23) | 1.27 (1.20-1.34) | 1.34 (1.27-1.42) |  |
|  | 4-highest | 13531 | 20.95 | 21.29 (20.65-21.92) | 1.47 (1.38-1.57) | 1.57 (1.47-1.67) |  |
| <40k | 1-lowest | 2083 | 21.27 | 21.67 (20.05-23.30) | 1.50 (1.31-1.72) | 1.61 (1.41-1.84) |  |
|  | 2 | 6002 | 19.44 | 19.68 (18.73-20.64) | 1.34 (1.22-1.47) | 1.42 (1.29-1.56) |  |
|  | 3 | 14351 | 20.06 | 20.47 (19.85-21.08) | 1.40 (1.31-1.49) | 1.50 (1.40-1.60) |  |
|  | 4-highest | 29737 | 22.63 | 23.40 (22.97-23.83) | 1.65 (1.57-1.73) | 1.81 (1.72-1.91) |  |
| Area-level income level*Denial rate | |  |  |  |  |  | 0.0097 |

Notes:

aRR = adjusted risk ratio; RR=risk ratio; Ref=reference category; PCT= percentage of patients who received guideline-concordant treatment; 95%IC = 95% confidence interval

^a^ Receipt of treatment modalities evaluated only among patients for whom it is recommended based on NCCN guidelines for NSCLC cancer.

^b^ p-value is for chi-square test

**Supplementary Table 4.** Sensitivity Analysis Examining Quartiles of Mortgage Denial Rates: Association of Area-Level Mortgage Denial Rate and Non-Guideline-Concordant NSCLC Care by Area-Level Income: ≥ 10 lymph nodes evaluated^a^

| Income level | Mortgage Denial rate | No. Patients | PCT (%) | Predicted Probabilities  (Adjusted for age and sex) | Crude RR (95%IC) | aRR (95%IC)  Adjusted for age and sex | P-value^b^ |
| --- | --- | --- | --- | --- | --- | --- | --- |
| ≥63k | 1-lowest | 23466 | 50.01 | 49.91 (49.27-50.55) | Ref | Ref |  |
|  | 2 | 13750 | 50.86 | 50.75 (49.91-51.58) | 1.04 (0.99-1.10) | 1.04 (0.99-1.10) |  |
|  | 3 | 7086 | 52.67 | 52.51 (51.34-53.67) | 1.10 (1.02-1.18) | 1.10 (1.02-1.17) |  |
|  | 4-highest | 1863 | 52.17 | 52.05 (49.78-54.31) | 1.08 (0.96-1.20) | 1.08 (0.96-1.20) |  |
| 50-63k | 1-lowest | 10999 | 51.11 | 51.08 (50.15-52.02) | 1.04 (0.98-1.11) | 1.05 (0.99-1.11) |  |
|  | 2 | 13802 | 50.75 | 50.76 (49.92-51.59) | 1.03 (0.97-1.09) | 1.03 (0.98-1.10) |  |
|  | 3 | 9768 | 50.66 | 50.66 (49.67-51.65) | 1.02 (0.96-1.08) | 1.02 (0.96-1.09) |  |
|  | 4-highest | 3802 | 52.21 | 52.22 (50.63-53.80) | 1.12 (1.02-1.22) | 1.12 (1.03-1.23) |  |
| 40-50k | 1-lowest | 4936 | 52.19 | 52.24 (50.84-53.63) | 1.09 (1.01-1.18) | 1.10 (1.02-1.19) |  |
|  | 2 | 10073 | 50.96 | 51.03 (50.05-52.00) | 1.05 (0.99-1.12) | 1.06 (0.99-1.12) |  |
|  | 3 | 11936 | 50.93 | 51.00 (50.10-51.89) | 1.03 (0.97-1.09) | 1.04 (0.98-1.10) |  |
|  | 4-highest | 6887 | 51.39 | 51.45 (50.27-52.63) | 1.09 (1.02-1.17) | 1.10 (1.03-1.18) |  |
| <40k | 1-lowest | 922 | 49.57 | 49.79 (46.57-53.02) | 1.03 (0.88-1.21) | 1.04 (0.89-1.22) |  |
|  | 2 | 2742 | 53.79 | 53.92 (52.05-55.79) | 1.16 (1.05-1.29) | 1.17 (1.06-1.30) |  |
|  | 3 | 6339 | 52.74 | 52.88 (51.66-54.11) | 1.11 (1.03-1.19) | 1.12 (1.04-1.20) |  |
|  | 4-highest | 13432 | 53.18 | 53.32 (52.47-54.16) | 1.14 (1.08-1.21) | 1.16 (1.10-1.22) |  |
| Area-level income level*Denial rate | |  |  |  |  |  | 0.8301 |

Notes:

aRR = adjusted risk ratio; RR=risk ratio; Ref=reference category; PCT= percentage of patients who received guideline-concordant treatment; 95%IC = 95% confidence interval

^a^ Receipt of treatment modalities evaluated only among patients for whom it is recommended based on NCCN guidelines for NSCLC cancer.

^b^ p-value is for chi-square test

**Supplementary Table 5.** Sensitivity Analysis Examining Quartiles of Mortgage Denial Rates: Association of Area-Level Mortgage Denial Rate and Non-Guideline-Concordant NSCLC Care by Area-Level Income: Neoadjuvant Chemoradiation^a^

| Income level | Mortgage Denial rate | No. Patients | PCT (%) | Predicted Probabilities  (Adjusted for age and sex) | Crude RR (95%IC) | aRR (95%IC)  Adjusted for age and sex | P-value^b^ |
| --- | --- | --- | --- | --- | --- | --- | --- |
| ≥63k | 1-lowest | 11309 | 26.73 | 25.63 (24.82-26.45) | Ref | Ref |  |
|  | 2 | 7097 | 27.35 | 26.55 (25.52-27.57) | 1.03 (0.96-1.11) | 1.05 (0.98-1.13) |  |
|  | 3 | 3251 | 30.51 | 29.53 (28.02-31.05) | 1.21 (1.10-1.32) | 1.22 (1.12-1.34) |  |
|  | 4-highest | 748 | 30.75 | 30.46 (27.31-33.62) | 1.22 (1.03-1.45) | 1.28 (1.07-1.53) |  |
| 50-63k | 1-lowest | 6722 | 26.45 | 25.89 (24.84-26.95) | 0.98 (0.91-1.05) | 1.01 (0.94-1.09) |  |
|  | 2 | 8972 | 26.71 | 26.66 (25.75-27.57) | 1.00 (0.94-1.07) | 1.06 (0.99-1.14) |  |
|  | 3 | 6120 | 28.42 | 28.28 (27.18-29.38) | 1.09 (1.01-1.18) | 1.15 (1.07-1.25) |  |
|  | 4-highest | 2135 | 28.95 | 29.30 (27.43-31.17) | 1.10 (0.99-1.23) | 1.20 (1.07-1.33) |  |
| 40-50k | 1-lowest | 3205 | 26.33 | 26.53 (25.01-28.06) | 0.98 (0.89-1.08) | 1.05 (0.95-1.16) |  |
|  | 2 | 6981 | 26.94 | 27.20 (26.17-28.24) | 1.01 (0.94-1.09) | 1.09 (1.01-1.17) |  |
|  | 3 | 8616 | 28.37 | 28.60 (27.67-29.53) | 1.09 (1.01-1.16) | 1.17 (1.09-1.26) |  |
|  | 4-highest | 4752 | 28.77 | 29.29 (28.03-30.54) | 1.10 (1.01-1.20) | 1.21 (1.11-1.32) |  |
| <40k | 1-lowest | 742 | 29.65 | 29.95 (26.78-33.12) | 1.15 (0.98-1.34) | 1.24 (1.05-1.46) |  |
|  | 2 | 2188 | 27.47 | 28.14 (26.29-29.98) | 1.06 (0.95-1.18) | 1.16 (1.04-1.30) |  |
|  | 3 | 5318 | 28.19 | 28.82 (27.64-30.01) | 1.07 (0.99-1.16) | 1.18 (1.09-1.28) |  |
|  | 4-highest | 11597 | 29.37 | 30.55 (29.75-31.36) | 1.15 (1.08-1.23) | 1.30 (1.22-1.39) |  |
| Area-level income level*Denial rate | |  |  |  |  |  | 0.3880 |

Notes:

aRR = adjusted risk ratio; RR=risk ratio; Ref=reference category; PCT= percentage of patients who received guideline-concordant treatment; 95%IC = 95% confidence interval

^a^ Receipt of treatment modalities evaluated only among patients for whom it is recommended based on NCCN guidelines for NSCLC cancer.

^b^ p-value is for chi-square test

**Supplementary Table 6.** Sensitivity Analysis Examining Quartiles of Mortgage Denial Rates: Association of Area-Level Mortgage Denial Rate and Non-Guideline-Concordant NSCLC Care by Area-Level Income: Chemotherapy or Immunotherapy^a^

| Income level | Mortgage Denial rate | No. Patients | PCT (%) | Predicted Probabilities  (Adjusted for age and sex) | Crude RR (95%IC) | aRR (95%IC)  Adjusted for age and sex | P-value^b^ |
| --- | --- | --- | --- | --- | --- | --- | --- |
| ≥63k | 1-lowest | 38802 | 49.08 | 47.33 (46.85-47.81) | Ref | Ref |  |
|  | 2 | 23904 | 49.23 | 47.68 (47.07-48.29) | 1.01 (0.97-1.04) | 1.01 (0.98-1.05) |  |
|  | 3 | 11262 | 51.47 | 49.89 (49.00-50.78) | 1.10 (1.05-1.15) | 1.11 (1.06-1.17) |  |
|  | 4-highest | 2791 | 51.16 | 49.63 (47.85-51.41) | 1.07 (0.98-1.17) | 1.08 (0.99-1.19) |  |
| 50-63k | 1-lowest | 21589 | 50.15 | 49.34 (48.70-49.98) | 1.04 (1.00-1.08) | 1.09 (1.05-1.14) |  |
|  | 2 | 28105 | 50.22 | 50.23 (49.67-50.79) | 1.05 (1.01-1.09) | 1.14 (1.10-1.18) |  |
|  | 3 | 19401 | 50.86 | 50.97 (50.29-51.65) | 1.08 (1.03-1.12) | 1.18 (1.13-1.23) |  |
|  | 4-highest | 7500 | 51.72 | 51.84 (50.75-52.93) | 1.11 (1.05-1.17) | 1.21 (1.14-1.29) |  |
| 40-50k | 1-lowest | 9971 | 51.32 | 51.10 (50.16-52.04) | 1.10 (1.04-1.16) | 1.18 (1.12-1.25) |  |
|  | 2 | 21702 | 51.93 | 52.42 (51.79-53.06) | 1.12 (1.08-1.17) | 1.25 (1.20-1.30) |  |
|  | 3 | 26154 | 52.00 | 52.59 (52.01-53.18) | 1.12 (1.08-1.17) | 1.26 (1.21-1.31) |  |
|  | 4-highest | 15281 | 53.26 | 54.23 (53.47-54.99) | 1.19 (1.13-1.24) | 1.36 (1.29-1.42) |  |
| <40k | 1-lowest | 2332 | 54.93 | 55.76 (53.81-57.71) | 1.25 (1.13-1.39) | 1.43 (1.28-1.59) |  |
|  | 2 | 6414 | 54.04 | 54.92 (53.74-56.09) | 1.22 (1.15-1.30) | 1.39 (1.30-1.49) |  |
|  | 3 | 15919 | 53.14 | 54.37 (53.63-55.12) | 1.18 (1.13-1.23) | 1.37 (1.31-1.43) |  |
|  | 4-highest | 35528 | 54.33 | 56.44 (55.94-56.94) | 1.25 (1.21-1.29) | 1.51 (1.45-1.56) |  |
| Area-level income level*Denial rate | |  |  |  |  |  | 0.5096 |

Notes:

aRR = adjusted risk ratio; RR=risk ratio; Ref=reference category; PCT= percentage of patients who received guideline-concordant treatment; 95%IC = 95% confidence interval

^a^ Receipt of treatment modalities evaluated only among patients for whom it is recommended based on NCCN guidelines for NSCLC cancer.

^b^ p-value is for chi-square test

**Supplementary Table 7.** Sensitivity Analysis Examining Association of Area-Level Mortgage Denial Rates and Time from Surgery to Chemotherapy Initiation by Area-Level Income

| Income level | Mortgage Denial rate | No. Patients | Crude HR (95%IC) | aHR (95%IC)  Adjusted for age and sex | P-value^a^ |
| --- | --- | --- | --- | --- | --- |
| ≥63k | 1-lowest | 90115 | Ref | Ref |  |
|  | 2 | 53866 | 1.06 (1.03-1.09) | 1.06 (1.03-1.08) |  |
|  | 3 | 25654 | 1.19 (1.15-1.23) | 1.18 (1.14-1.22) |  |
|  | 4-highest | 6314 | 1.19 (1.13-1.25) | 1.18 (1.12-1.24) |  |
| 50-63k | 1-lowest | 48794 | 0.99 (0.96-1.02) | 0.99 (0.97-1.02) |  |
|  | 2 | 62160 | 1.03 (1.00-1.06) | 1.04 (1.01-1.07) |  |
|  | 3 | 42459 | 1.06 (1.03-1.09) | 1.07 (1.04-1.10) |  |
|  | 4-highest | 15809 | 1.15 (1.10-1.20) | 1.15 (1.10-1.21) |  |
| 40-50k | 1-lowest | 22490 | 1.01 (0.97-1.04) | 1.02 (0.98-1.06) |  |
|  | 2 | 47713 | 1.08 (1.05-1.11) | 1.10 (1.07-1.13) |  |
|  | 3 | 56631 | 1.07 (1.04-1.10) | 1.09 (1.06-1.12) |  |
|  | 4-highest | 31968 | 1.09 (1.05-1.12) | 1.10 (1.07-1.14) |  |
| <40k | 1-lowest | 4895 | 1.03 (0.96-1.11) | 1.06 (0.99-1.15) |  |
|  | 2 | 13805 | 1.11 (1.06-1.16) | 1.13 (1.08-1.18) |  |
|  | 3 | 33760 | 1.08 (1.04-1.12) | 1.11 (1.07-1.15) |  |
|  | 4-highest | 72855 | 1.10 (1.07-1.13) | 1.13 (1.11-1.16) |  |
| Area-level income level*Denial rate | |  |  |  | <0.0001 |

Notes:

aHR = adjusted hazard ratio; HR= hazard ratio; Ref=reference category; 95%IC = 95% confidence interval

^a^ p-value is for chi-square test

**Supplementary Table 8.** Sensitivity Analysis Examining Association of Area-Level Mortgage Denial Rates and Overall Survival (Age as Time Scale) by Area-Level Income

| Income level | Mortgage Denial rate | No. Patients | Crude HR (95%IC) | aHR (95%IC)  Adjusted for age and sex | P-value^a^ |
| --- | --- | --- | --- | --- | --- |
| ≥63k | 1-lowest | 90115 | Ref | Ref |  |
|  | 2 | 53866 | 1.02 (1.00-1.04) | 1.02 (1.00-1.03) |  |
|  | 3 | 25654 | 0.95 (0.93-0.98) | 0.96 (0.93-0.98) |  |
|  | 4-highest | 6314 | 0.86 (0.83-0.89) | 0.86 (0.83-0.89) |  |
| 50-63k | 1-lowest | 48794 | 1.10 (1.09-1.12) | 1.10 (1.08-1.12) |  |
|  | 2 | 62160 | 1.13 (1.11-1.14) | 1.12 (1.11-1.14) |  |
|  | 3 | 42459 | 1.10 (1.08-1.12) | 1.10 (1.08-1.12) |  |
|  | 4-highest | 15809 | 0.99 (0.96-1.03) | 0.99 (0.95-1.02) |  |
| 40-50k | 1-lowest | 22490 | 1.16 (1.13-1.18) | 1.15 (1.13-1.17) |  |
|  | 2 | 47713 | 1.18 (1.16-1.19) | 1.17 (1.15-1.19) |  |
|  | 3 | 56631 | 1.19 (1.17-1.20) | 1.17 (1.16-1.19) |  |
|  | 4-highest | 31968 | 1.12 (1.09-1.14) | 1.10 (1.08-1.13) |  |
| <40k | 1-lowest | 4895 | 1.20 (1.15-1.24) | 1.18 (1.14-1.23) |  |
|  | 2 | 13805 | 1.24 (1.21-1.27) | 1.22 (1.19-1.25) |  |
|  | 3 | 33760 | 1.23 (1.21-1.26) | 1.22 (1.20-1.24) |  |
|  | 4-highest | 72855 | 1.23 (1.21-1.25) | 1.21 (1.20-1.23) |  |
| Area-level income level*Denial rate | |  |  |  | 0.1799 |

Notes:

aHR = adjusted hazard ratio; HR= hazard ratio; Ref=reference category; 95%IC = 95% confidence interval

^a^ p-value is for chi-square test

**Supplementary Table 9.** Sensitivity Analysis Examining Racial Differences in Association of Area-Level Mortgage Denial Rate and Non-Guideline-Concordant NSCLC Care: Overall Guideline-Concordant Care

|  |  | **All Guideline-Concordant Care** | | | | | |  |
| --- | --- | --- | --- | --- | --- | --- | --- | --- |
| Race /Ethnicity | Mortgage Denial Rate | Yes | | No | | Crude Risk Ratio | Adjusted Risk Ratio  (Adjusted for age + sex + income) | p-value^a^ |
|  |  | N | (%) | N | (%) | (95% CI) |  |  |
| NH White | 1-lowest | 51333 | 53.07 | 45387 | 46.93 | Ref | Ref |  |
|  | 2 | 50333 | 52.67 | 45223 | 47.33 | 1.01 (1.00-1.02) | 1.02 (1.01-1.03) |  |
|  | 3 | 48168 | 51.74 | 44928 | 48.26 | 1.03 (1.02-1.04) | 1.04 (1.03-1.05) |  |
|  | 4 | 45405 | 51.12 | 43410 | 48.88 | 1.04 (1.03-1.06) | 1.05 (1.04-1.07) |  |
|  | 5-highest | 32169 | 49.50 | 32814 | 50.50 | 1.08 (1.06-1.09) | 1.09 (1.08-1.11) |  |
| Hispanic | 1-lowest | 1017 | 48.78 | 1068 | 51.22 | 1.06 (1.02-1.11) | 1.10 (1.05-1.15) |  |
|  | 2 | 1104 | 46.15 | 1288 | 53.85 | 1.12 (1.07-1.16) | 1.16 (1.11-1.20) |  |
|  | 3 | 1439 | 46.78 | 1637 | 53.22 | 1.10 (1.06-1.14) | 1.14 (1.10-1.18) |  |
|  | 4 | 1732 | 46.26 | 2012 | 53.74 | 1.12 (1.09-1.16) | 1.16 (1.13-1.20) |  |
|  | 5-highest | 3445 | 44.69 | 4264 | 55.31 | 1.16 (1.13-1.19) | 1.19 (1.16-1.22) |  |
| NH Black | 1-lowest | 2194 | 49.80 | 2212 | 50.20 | 1.06 (1.03-1.10) | 1.12 (1.09-1.16) |  |
|  | 2 | 3115 | 50.73 | 3025 | 49.27 | 1.05 (1.02-1.08) | 1.10 (1.07-1.14) |  |
|  | 3 | 3900 | 50.45 | 3831 | 49.55 | 1.05 (1.03-1.08) | 1.10 (1.08-1.13) |  |
|  | 4 | 6034 | 49.75 | 6094 | 50.25 | 1.06 (1.04-1.09) | 1.11 (1.09-1.14) |  |
|  | 5-highest | 15528 | 48.74 | 16328 | 51.26 | 1.09 (1.08-1.11) | 1.14 (1.13-1.16) |  |
| NH Asian & PI | 1-lowest | 1933 | 54.64 | 1605 | 45.36 | 0.96 (0.92-0.99) | 0.99 (0.96-1.03) |  |
|  | 2 | 1701 | 52.47 | 1541 | 47.53 | 1.01 (0.97-1.05) | 1.04 (1.00-1.08) |  |
|  | 3 | 1916 | 53.24 | 1683 | 46.76 | 0.97 (0.93-1.01) | 1.00 (0.97-1.04) |  |
|  | 4 | 1493 | 51.57 | 1402 | 48.43 | 1.01 (0.96-1.05) | 1.04 (1.00-1.08) |  |
|  | 5-highest | 1375 | 49.91 | 1380 | 50.09 | 1.04 (0.99-1.09) | 1.09 (1.05-1.14) |  |
| NH Other | 1-lowest | 439 | 51.77 | 409 | 48.23 | 1.02 (0.95-1.09) | 1.07 (1.00-1.15) |  |
|  | 2 | 377 | 50.67 | 367 | 49.33 | 1.04 (0.97-1.13) | 1.08 (1.00-1.16) |  |
|  | 3 | 338 | 44.89 | 415 | 55.11 | 1.16 (1.08-1.24) | 1.20 (1.12-1.28) |  |
|  | 4 | 365 | 46.14 | 426 | 53.86 | 1.13 (1.06-1.21) | 1.18 (1.11-1.26) |  |
|  | 5-highest | 547 | 46.08 | 640 | 53.92 | 1.14 (1.08-1.20) | 1.19 (1.12-1.25) |  |
| Race/Ethnicity * Denial rate levels |  |  |  |  |  |  |  | 0.9989 |

Notes:

CI = confidence interval; NH=Non-Hispanic; NH Asian & PI = Non-Hispanic Asian and Pacific Islander; NSCLC = non-small cell lung cancer

^a^ p-value is for chi-square test

**Supplementary Table 10.** Sensitivity Analysis Examining Racial Differences in Association of Area-Level Mortgage Denial Rate and Non-Guideline-Concordant NSCLC Care: Surgery

|  |  | **Surgery** | | | | | |  |
| --- | --- | --- | --- | --- | --- | --- | --- | --- |
| Race /Ethnicity | Denial Rate Level | Yes | | No | | Crude Risk Ratio | Adjusted Risk Ratio  (Adjusted for age + sex + income) | p-value^a^ |
|  |  | N | (%) | N | (%) | (95% CI) |  |  |
| NH White | 1-lowest | 44672 | 84.77 | 8023 | 15.23 | Ref | Ref |  |
|  | 2 | 42715 | 83.82 | 8243 | 16.18 | 1.07 (1.03-1.11) | 1.07 (1.03-1.11) |  |
|  | 3 | 40734 | 82.83 | 8443 | 17.17 | 1.13 (1.09-1.17) | 1.12 (1.07-1.17) |  |
|  | 4 | 38326 | 82.25 | 8272 | 17.75 | 1.17 (1.13-1.21) | 1.15 (1.10-1.20) |  |
|  | 5-highest | 26799 | 80.05 | 6680 | 19.95 | 1.32 (1.27-1.37) | 1.31 (1.25-1.37) |  |
| Hispanic | 1-lowest | 795 | 77.71 | 228 | 22.29 | 1.35 (1.18-1.55) | 1.57 (1.33-1.86) |  |
|  | 2 | 850 | 76.51 | 261 | 23.49 | 1.45 (1.30-1.62) | 1.67 (1.44-1.93) |  |
|  | 3 | 1065 | 75.64 | 343 | 24.36 | 1.50 (1.35-1.67) | 1.71 (1.49-1.96) |  |
|  | 4 | 1265 | 75.30 | 415 | 24.70 | 1.56 (1.42-1.72) | 1.79 (1.58-2.03) |  |
|  | 5-highest | 2939 | 76.72 | 892 | 23.28 | 1.55 (1.43-1.68) | 1.63 (1.46-1.82) |  |
| NH Black | 1-lowest | 1635 | 78.57 | 446 | 21.43 | 1.38 (1.26-1.51) | 1.73 (1.54-1.95) |  |
|  | 2 | 2241 | 78.58 | 611 | 21.42 | 1.39 (1.29-1.50) | 1.69 (1.53-1.87) |  |
|  | 3 | 2691 | 76.89 | 809 | 23.11 | 1.47 (1.37-1.58) | 1.78 (1.62-1.96) |  |
|  | 4 | 4213 | 76.21 | 1315 | 23.79 | 1.54 (1.45-1.63) | 1.81 (1.68-1.96) |  |
|  | 5-highest | 10437 | 74.90 | 3497 | 25.10 | 1.65 (1.58-1.72) | 1.89 (1.78-2.01) |  |
| NH Asian & PI | 1-lowest | 1318 | 83.63 | 258 | 16.37 | 1.05 (0.94-1.18) | 1.21 (1.06-1.39) |  |
|  | 2 | 1184 | 81.21 | 274 | 18.79 | 1.18 (1.06-1.32) | 1.34 (1.16-1.54) |  |
|  | 3 | 1252 | 78.20 | 349 | 21.80 | 1.37 (1.24-1.51) | 1.50 (1.32-1.71) |  |
|  | 4 | 1010 | 78.17 | 282 | 21.83 | 1.43 (1.29-1.58) | 1.52 (1.33-1.74) |  |
|  | 5-highest | 1077 | 81.34 | 247 | 18.66 | 1.25 (1.10-1.42) | 1.35 (1.16-1.57) |  |
| NH Other | 1-lowest | 315 | 82.46 | 67 | 17.54 | 1.10 (0.88-1.38) | 1.31 (1.00-1.72) |  |
|  | 2 | 272 | 79.30 | 71 | 20.70 | 1.32 (1.06-1.64) | 1.52 (1.14-2.01) |  |
|  | 3 | 306 | 79.27 | 80 | 20.73 | 1.33 (1.09-1.61) | 1.53 (1.18-1.97) |  |
|  | 4 | 312 | 81.25 | 72 | 18.75 | 1.21 (0.98-1.50) | 1.33 (1.02-1.75) |  |
|  | 5-highest | 444 | 76.82 | 134 | 23.18 | 1.51 (1.30-1.76) | 1.73 (1.41-2.12) |  |
| Race/Ethnicity * Denial Rate Level |  |  |  |  |  |  |  | 0.9469 |

Notes:

CI = confidence interval; NH=Non-Hispanic; NH Asian & PI = Non-Hispanic Asian and Pacific Islander; NSCLC = non-small cell lung cancer

^a^ p-value is for chi-square test

**Supplementary Table 11.** Sensitivity Analysis Examining Racial Differences in Association of Area-Level Mortgage Denial Rate and Non-Guideline-Concordant NSCLC Care: ≥ 10 lymph nodes evaluated

|  |  | **≥ 10 Lymph Nodes Evaluated** | | | | | |  |
| --- | --- | --- | --- | --- | --- | --- | --- | --- |
| Race /Ethnicity | Denial Rate Level | Yes | | No | | Crude Risk Ratio | Adjusted Risk Ratio  (Adjusted for age + sex + income) | p-value^a^ |
|  |  | N | (%) | N | (%) | (95% CI) |  |  |
| NH White | 1-lowest | 13857 | 49.41 | 14190 | 50.59 | Ref | Ref |  |
|  | 2 | 13319 | 49.96 | 13338 | 50.04 | 1.00 (0.97-1.02) | 0.99 (0.97-1.02) |  |
|  | 3 | 12358 | 48.82 | 12957 | 51.18 | 1.01 (0.99-1.03) | 1.01 (0.99-1.03) |  |
|  | 4 | 11456 | 48.76 | 12038 | 51.24 | 1.01 (0.99-1.03) | 1.01 (0.99-1.03) |  |
|  | 5-highest | 8002 | 48.69 | 8431 | 51.31 | 1.02 (1.00-1.04) | 1.02 (0.99-1.04) |  |
| Hispanic | 1-lowest | 268 | 46.13 | 313 | 53.87 | 1.06 (0.98-1.15) | 1.06 (0.98-1.15) |  |
|  | 2 | 291 | 46.26 | 338 | 53.74 | 1.04 (0.96-1.12) | 1.04 (0.97-1.12) |  |
|  | 3 | 366 | 46.27 | 425 | 53.73 | 1.04 (0.97-1.12) | 1.04 (0.97-1.12) |  |
|  | 4 | 404 | 45.91 | 476 | 54.09 | 1.07 (1.00-1.15) | 1.07 (1.00-1.15) |  |
|  | 5-highest | 1101 | 47.97 | 1194 | 52.03 | 1.05 (1.01-1.10) | 1.05 (1.00-1.10) |  |
| NH Black | 1-lowest | 428 | 44.12 | 542 | 55.88 | 1.11 (1.05-1.18) | 1.12 (1.05-1.19) |  |
|  | 2 | 588 | 43.82 | 754 | 56.18 | 1.12 (1.07-1.18) | 1.12 (1.07-1.18) |  |
|  | 3 | 704 | 44.76 | 869 | 55.24 | 1.10 (1.05-1.15) | 1.10 (1.05-1.15) |  |
|  | 4 | 1073 | 43.92 | 1370 | 56.08 | 1.10 (1.06-1.15) | 1.10 (1.05-1.15) |  |
|  | 5-highest | 2642 | 43.62 | 3415 | 56.38 | 1.13 (1.09-1.16) | 1.12 (1.08-1.15) |  |
| NH Asian & PI | 1-lowest | 530 | 51.36 | 502 | 48.64 | 0.98 (0.92-1.04) | 0.98 (0.92-1.05) |  |
|  | 2 | 416 | 46.64 | 476 | 53.36 | 1.05 (0.99-1.13) | 1.06 (0.99-1.13) |  |
|  | 3 | 482 | 49.33 | 495 | 50.67 | 0.99 (0.92-1.06) | 0.99 (0.92-1.06) |  |
|  | 4 | 419 | 52.9 | 373 | 47.1 | 0.93 (0.85-1.01) | 0.93 (0.85-1.01) |  |
|  | 5-highest | 496 | 53.16 | 437 | 46.84 | 0.97 (0.90-1.05) | 0.97 (0.90-1.05) |  |
| NH Other | 1-lowest | 111 | 51.87 | 103 | 48.13 | 0.94 (0.82-1.08) | 0.95 (0.83-1.09) |  |
|  | 2 | 90 | 51.72 | 84 | 48.28 | 0.95 (0.82-1.10) | 0.95 (0.82-1.11) |  |
|  | 3 | 92 | 44.23 | 116 | 55.77 | 1.08 (0.95-1.22) | 1.09 (0.96-1.23) |  |
|  | 4 | 104 | 48.6 | 110 | 51.4 | 1.01 (0.89-1.16) | 1.01 (0.89-1.16) |  |
|  | 5-highest | 110 | 37.80 | 181 | 62.20 | 1.23 (1.13-1.35) | 1.24 (1.13-1.36) |  |
| Race/Ethnicity * Denial Rate Level |  |  |  |  |  |  |  | 0.0924 |

Notes:

CI = confidence interval; NH=Non-Hispanic; NH Asian & PI = Non-Hispanic Asian and Pacific Islander; NSCLC = non-small cell lung cancer

^a^ p-value is for chi-square test

**Supplementary Table 12.** Sensitivity Analysis Examining Racial Differences in Association of Area-Level Mortgage Denial Rate and Non-Guideline-Concordant NSCLC Care: Neoadjuvant Chemoradiation

|  |  | **Neoadjuvant Chemoradiation** | | | | | |  |
| --- | --- | --- | --- | --- | --- | --- | --- | --- |
| Race /Ethnicity | Denial Rate Level | Yes | | No | | Crude Risk Ratio | Adjusted Risk Ratio  (Adjusted for age + sex + income) | p-value^a^ |
|  |  | N | (%) | N | (%) | (95% CI) |  |  |
| NH White | 1-lowest | 10936 | 73.71 | 3901 | 26.29 | Ref | Ref |  |
|  | 2 | 11545 | 73.08 | 4252 | 26.92 | 1.03 (0.99-1.07) | 1.05 (1.00-1.11) |  |
|  | 3 | 11327 | 73.05 | 4178 | 26.95 | 1.03 (0.99-1.07) | 1.08 (1.02-1.14) |  |
|  | 4 | 10957 | 71.36 | 4397 | 28.64 | 1.09 (1.05-1.13) | 1.17 (1.11-1.24) |  |
|  | 5-highest | 8033 | 70.28 | 3397 | 29.72 | 1.13 (1.09-1.18) | 1.27 (1.19-1.35) |  |
| Hispanic | 1-lowest | 218 | 68.55 | 100 | 31.45 | 1.18 (0.99-1.39) | 1.35 (1.05-1.73) |  |
|  | 2 | 240 | 61.22 | 152 | 38.78 | 1.44 (1.26-1.66) | 1.84 (1.47-2.32) |  |
|  | 3 | 269 | 61.28 | 170 | 38.72 | 1.45 (1.28-1.65) | 1.86 (1.51-2.28) |  |
|  | 4 | 366 | 62.78 | 217 | 37.22 | 1.39 (1.24-1.56) | 1.69 (1.41-2.03) |  |
|  | 5-highest | 754 | 63.63 | 431 | 36.37 | 1.36 (1.26-1.48) | 1.66 (1.46-1.89) |  |
| NH Black | 1-lowest | 559 | 71.85 | 219 | 28.15 | 1.06 (0.95-1.19) | 1.24 (1.07-1.45) |  |
|  | 2 | 831 | 74.66 | 282 | 25.34 | 0.97 (0.87-1.08) | 1.10 (0.96-1.27) |  |
|  | 3 | 1105 | 74.86 | 371 | 25.14 | 0.96 (0.88-1.05) | 1.07 (0.94-1.22) |  |
|  | 4 | 1719 | 73.06 | 634 | 26.94 | 1.03 (0.96-1.10) | 1.16 (1.05-1.29) |  |
|  | 5-highest | 4475 | 73.11 | 1646 | 26.89 | 1.03 (0.98-1.08) | 1.18 (1.09-1.28) |  |
| NH Asian & PI | 1-lowest | 355 | 70.44 | 149 | 29.56 | 1.11 (0.96-1.28) | 1.22 (0.99-1.49) |  |
|  | 2 | 301 | 66.89 | 149 | 33.11 | 1.25 (1.09-1.44) | 1.40 (1.14-1.72) |  |
|  | 3 | 396 | 74.16 | 138 | 25.84 | 0.98 (0.85-1.13) | 0.99 (0.82-1.19) |  |
|  | 4 | 294 | 71.01 | 120 | 28.99 | 1.11 (0.94-1.30) | 1.22 (0.97-1.54) |  |
|  | 5-highest | 257 | 71.59 | 102 | 28.41 | 1.05 (0.88-1.25) | 1.15 (0.91-1.46) |  |
| NH Other | 1-lowest | 108 | 70.13 | 46 | 29.87 | 1.14 (0.89-1.45) | 1.38 (0.97-1.98) |  |
|  | 2 | 87 | 64.93 | 47 | 35.07 | 1.34 (1.07-1.69) | 1.63 (1.14-2.32) |  |
|  | 3 | 78 | 66.67 | 39 | 33.33 | 1.27 (0.98-1.65) | 1.60 (1.06-2.43) |  |
|  | 4 | 85 | 67.46 | 41 | 32.54 | 1.23 (0.96-1.58) | 1.59 (1.09-2.32) |  |
|  | 5-highest | 139 | 69.50 | 61 | 30.50 | 1.17 (0.95-1.43) | 1.43 (1.06-1.93) |  |
| Race/Ethnicity * Denial Rate Level |  |  |  |  |  |  |  | <0.0001 |

Notes:

CI = confidence interval; NH=Non-Hispanic; NH Asian & PI = Non-Hispanic Asian and Pacific Islander; NSCLC = non-small cell lung cancer

^a^ p-value is for chi-square test

**Supplementary Table 13.** Sensitivity Analysis Examining Racial Differences in Association of Area-Level Mortgage Denial Rate and Non-Guideline-Concordant NSCLC Care: Chemotherapy or Immunotherapy

|  |  | **Chemotherapy or Immunotherapy** | | | | | |  |
| --- | --- | --- | --- | --- | --- | --- | --- | --- |
| Race /Ethnicity | Denial Rate Level | Yes | | No | | Crude Risk Ratio | Adjusted Risk Ratio  (Adjusted for age + sex + income) | p-value^a^ |
|  |  | N | (%) | N | (%) | (95% CI) |  |  |
| NH White | 1-lowest | 24590 | 48.04 | 26594 | 51.96 | Ref | Ref |  |
|  | 2 | 24467 | 47.53 | 27013 | 52.47 | 1.01 (1.00-1.03) | 1.03 (1.00-1.06) |  |
|  | 3 | 23916 | 46.84 | 27138 | 53.16 | 1.02 (1.01-1.04) | 1.06 (1.03-1.09) |  |
|  | 4 | 22445 | 45.90 | 26459 | 54.10 | 1.04 (1.03-1.06) | 1.10 (1.07-1.13) |  |
|  | 5-highest | 16081 | 44.15 | 20343 | 55.85 | 1.08 (1.06-1.09) | 1.20 (1.15-1.24) |  |
| Hispanic | 1-lowest | 608 | 47.39 | 675 | 52.61 | 1.00 (0.95-1.06) | 1.11 (0.98-1.24) |  |
|  | 2 | 675 | 45.98 | 793 | 54.02 | 1.02 (0.97-1.08) | 1.16 (1.04-1.30) |  |
|  | 3 | 919 | 47.10 | 1032 | 52.90 | 1.00 (0.96-1.05) | 1.12 (1.02-1.22) |  |
|  | 4 | 1086 | 45.61 | 1295 | 54.39 | 1.04 (1.00-1.08) | 1.19 (1.09-1.30) |  |
|  | 5-highest | 2036 | 43.99 | 2592 | 56.01 | 1.07 (1.04-1.11) | 1.22 (1.13-1.31) |  |
| NH Black | 1-lowest | 1266 | 47.47 | 1401 | 52.53 | 1.00 (0.96-1.04) | 1.23 (1.12-1.34) |  |
|  | 2 | 1771 | 48.51 | 1880 | 51.49 | 0.98 (0.95-1.02) | 1.13 (1.05-1.22) |  |
|  | 3 | 2279 | 48.39 | 2431 | 51.61 | 0.99 (0.96-1.02) | 1.13 (1.06-1.21) |  |
|  | 4 | 3496 | 47.51 | 1551 | 52.49 | 1.00 (0.98-1.03) | 1.15 (1.09-1.22) |  |
|  | 5-highest | 9265 | 46.40 | 4129 | 53.60 | 1.03 (1.01-1.05) | 1.18 (1.13-1.23) |  |
| NH Asian & PI | 1-lowest | 1193 | 55.70 | 362 | 44.30 | 0.84 (0.80-0.89) | 0.82 (0.75-0.90) |  |
|  | 2 | 1086 | 54.85 | 345 | 45.15 | 0.86 (0.82-0.91) | 0.83 (0.75-0.92) |  |
|  | 3 | 1225 | 55.06 | 399 | 44.94 | 0.86 (0.81-0.90) | 0.77 (0.70-0.85) |  |
|  | 4 | 929 | 51.84 | 319 | 48.16 | 0.91 (0.86-0.97) | 0.88 (0.79-0.97) |  |
|  | 5-highest | 803 | 48.52 | 348 | 51.48 | 0.97 (0.92-1.03) | 1.01 (0.90-1.15) |  |
| NH Other | 1-lowest | 249 | 48.54 | 107 | 51.46 | 0.99 (0.90-1.08) | 1.20 (0.99-1.45) |  |
|  | 2 | 196 | 45.48 | 90 | 54.52 | 1.05 (0.96-1.15) | 1.19 (0.97-1.45) |  |
|  | 3 | 203 | 44.42 | 108 | 55.58 | 1.06 (0.97-1.15) | 1.26 (1.03-1.54) |  |
|  | 4 | 197 | 41.91 | 109 | 58.09 | 1.11 (1.03-1.20) | 1.45 (1.21-1.75) |  |
|  | 5-highest | 332 | 45.67 | 173 | 54.33 | 1.05 (0.98-1.12) | 1.24 (1.06-1.46) |  |
| Race/Ethnicity * Denial Rate Level |  |  |  |  |  |  |  | 0.1816 |

Notes:

CI = confidence interval; NH=Non-Hispanic; NH Asian & PI = Non-Hispanic Asian and Pacific Islander; NSCLC = non-small cell lung cancer

^a^ p-value is for chi-square test

**Supplementary Table 14.** Sensitivity Analysis Examining Racial Differences in Association of Area-Level Mortgage Denial Rate and Time from Surgery to Chemotherapy Initiation

|  |  | **Time to Chemotherapy Initiation** | |  |
| --- | --- | --- | --- | --- |
| Race /Ethnicity | Denial Rate Level | Crude Hazard Ratio | Adjusted Hazard Ratio  (Adjusted for age + sex + income) | p-value^a^ |
|  |  | (95% CI) | (95% CI) |  |
| NH White | 1-lowest | Ref | Ref |  |
|  | 2 | 1.03 (1.01-1.04) | 1.02 (1.00-1.03) |  |
|  | 3 | 1.06 (1.04-1.07) | 1.04 (1.02-1.05) |  |
|  | 4 | 1.08 (1.06-1.09) | 1.05 (1.03-1.06) |  |
|  | 5-highest | 1.11 (1.09-1.13) | 1.07 (1.05-1.09) |  |
| Hispanic | 1-lowest | 0.96 (0.90-1.02) | 1.01 (0.95-1.08) |  |
|  | 2 | 0.99 (0.93-1.06) | 1.02 (0.95-1.09) |  |
|  | 3 | 1.08 (1.02-1.14) | 1.10 (1.03-1.16) |  |
|  | 4 | 1.06 (1.00-1.13) | 1.07 (1.01-1.14) |  |
|  | 5-highest | 1.14 (1.09-1.18) | 1.09 (1.05-1.13) |  |
| NH Black | 1-lowest | 1.02 (0.96-1.08) | 1.07 (1.01-1.13) |  |
|  | 2 | 0.99 (0.94-1.03) | 1.03 (0.99-1.08) |  |
|  | 3 | 1.01 (0.96-1.05) | 1.04 (0.99-1.08) |  |
|  | 4 | 1.03 (0.99-1.06) | 1.04 (1.00-1.08) |  |
|  | 5-highest | 1.06 (1.04-1.09) | 1.05 (1.03-1.08) |  |
| NH Asian & PI | 1-lowest | 0.88 (0.84-0.92) | 0.94 (0.89-0.99) |  |
|  | 2 | 0.89 (0.84-0.94) | 0.92 (0.88-0.97) |  |
|  | 3 | 0.90 (0.85-0.95) | 0.91 (0.86-0.96) |  |
|  | 4 | 0.94 (0.89-1.00) | 0.94 (0.88-1.00) |  |
|  | 5-highest | 1.04 (0.98-1.10) | 1.02 (0.96-1.08) |  |
| NH Other | 1-lowest | 1.04 (0.93-1.16) | 1.09 (0.98-1.22) |  |
|  | 2 | 1.04 (0.92-1.18) | 1.06 (0.94-1.19) |  |
|  | 3 | 1.06 (0.94-1.19) | 1.07 (0.95-1.20) |  |
|  | 4 | 1.18 (1.06-1.31) | 1.18 (1.06-1.31) |  |
|  | 5-highest | 1.17 (1.06-1.29) | 1.18 (1.07-1.31) |  |
| Race/Ethnicity * Denial Rate Level |  |  |  | 0.5161 |

Notes:

CI = confidence interval; NH=Non-Hispanic; NH Asian & PI = Non-Hispanic Asian and Pacific Islander

^a^ p-value is for chi-square test

**Supplementary Table 15.** Sensitivity Analysis Examining Racial Differences in Association of Area-Level Mortgage Denial Rate and Overall Survival

|  |  | **Overall survival** | |  |
| --- | --- | --- | --- | --- |
| Race /Ethnicity | Denial Rate Level | Crude Hazard Ratio | Adjusted Hazard Ratio  (Adjusted for sex + income) | p-value^b^ |
|  |  | (95% CI) | (95% CI) |  |
| NH White | 1-lowest | Ref | Ref |  |
|  | 2 | 1.04 (1.02-1.06) | 1.00 (0.98-1.01) |  |
|  | 3 | 1.07 (1.05-1.08) | 0.99 (0.98-1.01) |  |
|  | 4 | 1.08 (1.06-1.10) | 0.98 (0.96-0.99) |  |
|  | 5-highest | 1.11 (1.09-1.13) | 0.95 (0.94-0.97) |  |
| Hispanic | 1-lowest | 0.89 (0.84-0.95) | 0.89 (0.83-0.96) |  |
|  | 2 | 0.97 (0.91-1.03) | 0.93 (0.87-0.99) |  |
|  | 3 | 1.02 (0.96-1.07) | 0.94 (0.89-1.00) |  |
|  | 4 | 1.01 (0.95-1.07) | 0.90 (0.86-0.96) |  |
|  | 5-highest | 0.87 (0.83-0.91) | 0.74 (0.71-0.77) |  |
| NH Black | 1-lowest | 1.09 (1.04-1.14) | 1.07 (1.02-1.12) |  |
|  | 2 | 1.08 (1.04-1.12) | 1.02 (0.98-1.06) |  |
|  | 3 | 1.15 (1.12-1.19) | 1.05 (1.02-1.08) |  |
|  | 4 | 1.14 (1.10-1.17) | 1.01 (0.98-1.04) |  |
|  | 5-highest | 1.17 (1.14-1.19) | 0.98 (0.96-1.01) |  |
| NH Asian & PI | 1-lowest | 0.80 (0.77-0.84) | 0.86 (0.82-0.90) |  |
|  | 2 | 0.82 (0.78-0.87) | 0.84 (0.79-0.89) |  |
|  | 3 | 0.86 (0.81-0.92) | 0.84 (0.78-0.90) |  |
|  | 4 | 0.92 (0.86-0.99) | 0.86 (0.80-0.92) |  |
|  | 5-highest | 0.66 (0.59-0.74) | 0.58 (0.51-0.66) |  |
| NH Other | 1-lowest | 1.00 (0.91-1.11) | 1.03 (0.93-1.13) |  |
|  | 2 | 1.02 (0.92-1.14) | 0.99 (0.89-1.10) |  |
|  | 3 | 1.00 (0.91-1.11) | 0.93 (0.84-1.03) |  |
|  | 4 | 0.96 (0.87-1.07) | 0.87 (0.79-0.97) |  |
|  | 5-highest | 1.01 (0.93-1.11) | 0.86 (0.79-0.93) |  |
| Race/Ethnicity * Denial Rate Level |  |  |  | 0.2803 |

Notes:

CI = confidence interval; NH=Non-Hispanic; NH Asian & PI = Non-Hispanic Asian and Pacific Islander

^a^ p-value is for chi-square test
